# Repertoire of Intensive Care Unit Pneumonia Microbiota

**DOI:** 10.1371/journal.pone.0032486

**Published:** 2012-02-28

**Authors:** Sabri Bousbia, Laurent Papazian, Pierre Saux, Jean Marie Forel, Jean-Pierre Auffray, Claude Martin, Didier Raoult, Bernard La Scola

**Affiliations:** 1 URMITE, Unité de Recherche sur les Maladies Infectieuses et Tropicales Emergentes, Faculté de Médecine, CNRS-IRD UMR 6236, Marseille, France; 2 Service de Réanimation Médicale, Hôpital Nord, Marseille, France; 3 Département d'Anesthésie-Réanimation, Hôpital la Timone, Marseille, France; 4 Département d'Anesthésie-Réanimation, Hôpital Nord, Marseille, France; Columbia University, United States of America

## Abstract

Despite the considerable number of studies reported to date, the causative agents of pneumonia are not completely identified. We comprehensively applied modern and traditional laboratory diagnostic techniques to identify microbiota in patients who were admitted to or developed pneumonia in intensive care units (ICUs). During a three-year period, we tested the bronchoalveolar lavage (BAL) of patients with ventilator-associated pneumonia, community-acquired pneumonia, non-ventilator ICU pneumonia and aspiration pneumonia, and compared the results with those from patients without pneumonia (controls). Samples were tested by amplification of 16S rDNA, 18S rDNA genes followed by cloning and sequencing and by PCR to target specific pathogens. We also included culture, amoeba co-culture, detection of antibodies to selected agents and urinary antigen tests. Based on molecular testing, we identified a wide repertoire of 160 bacterial species of which 73 have not been previously reported in pneumonia. Moreover, we found 37 putative new bacterial phylotypes with a 16S rDNA gene divergence ≥98% from known phylotypes. We also identified 24 fungal species of which 6 have not been previously reported in pneumonia and 7 viruses. Patients can present up to 16 different microorganisms in a single BAL (mean ± SD; 3.77±2.93). Some pathogens considered to be typical for ICU pneumonia such as *Pseudomonas aeruginosa* and *Streptococcus* species can be detected as commonly in controls as in pneumonia patients which strikingly highlights the existence of a core pulmonary microbiota. Differences in the microbiota of different forms of pneumonia were documented.

## Introduction

The cause of pneumonia in intensive care units (ICUs) remains unknown in nearly 30% of cases despite extensive microbiological investigations [1], [2]. Microbial communities previously identified, in deep respiratory samples, include bacteria, fungi and viruses for which the role in the observed pathology is not clear. Microorganisms frequently identified in respiratory samples from ICU-pneumonia patients included *Pseudomonas aeruginosa*, Staphylococci, Enterobacteria, *Candida albicans*, Influenza virus, Herpes simplex virus (HSV) and Cytomegalovirus (CMV) [3]–[7]. In some investigations, a pathogenic bacterium is isolated, whereas in other cases, the number of colony forming units (CFU) is considered to determine the pathogenic character [8]. Recently, the bacterial microbiota of patients with cystic fibrosis and ventilator-associated pneumonia (VAP) were studied using 16S rDNA gene amplification followed by clone libraries sequencing [9]–[11]. Our laboratory has contributed to this work and has shown, by different sequencing approaches, that the microbial population of patients with cystic fibrosis was more diverse than expected [10], [11]. Here, we use a comparable approach in order to study 185 episodes of ICU pneumonia and 25 control cases. These patients were studied using broad-range primer amplification of the 16S rDNA gene of bacteria and the intergenic spacer of 18S rDNA gene of fungi followed by cloning and sequencing. We also used specific quantitative PCR (qPCR) to target fastidious bacteria and a spectrum of viruses. Moreover, we tested samples from our patients by standardized routine culture, amoebal co-culture, blood culture, ELISA targeted antibody detection, immunofluorescent assay antigenemia and antigenuria testing as routinely performed in such cases to compare these routine tests with molecular approaches.

In preliminary results, we have reported the likely frequency of *Tropheryma whipplei* and the occurrence of vegetable DNA in pneumonia patients [12], [13]. In this work, we highlight the different compositions of microbiota in patients with four different types of ICU-pneumonia.

## Results

### Bacterial microbiota as evaluated by 16S rDNA

**Figure 1:**
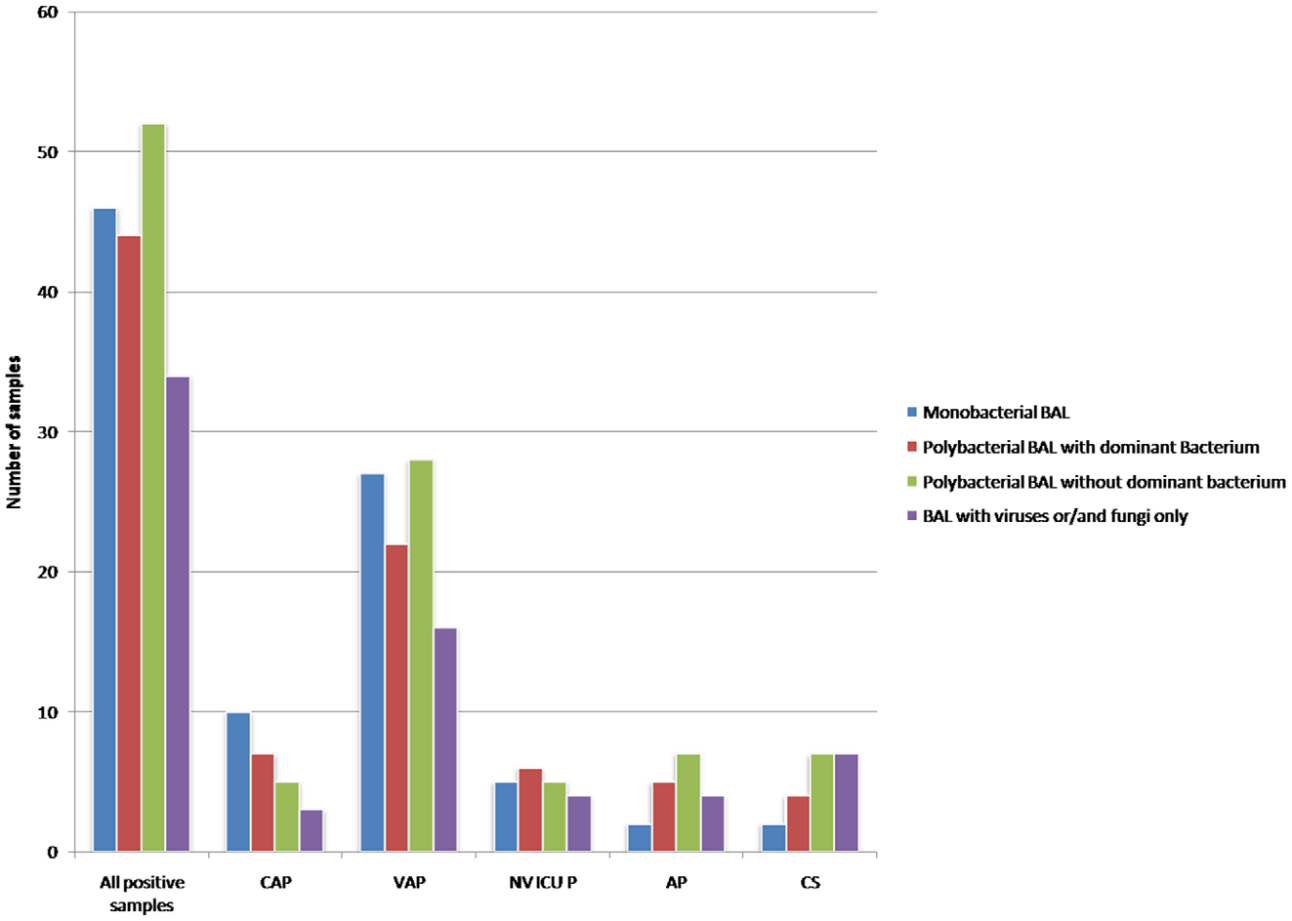
Microbial profiles of positive BAL fluids from patients and controls.

**Figure 2:**
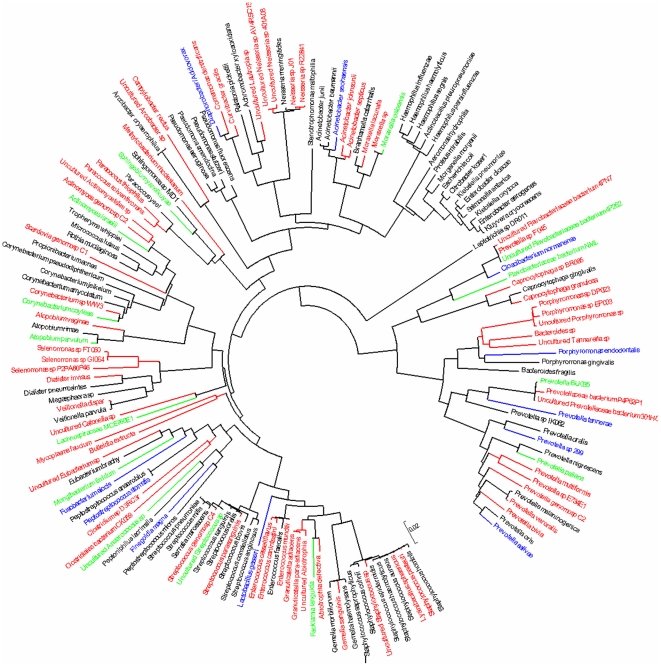
The phylogenetic tree inferred from bacterial 16S rDNA sequences which were identified in all patients using the Neighbor-Joining and the Kimura 2-parameter methods. Bacteria which were previously identified in pneumonia are shown in black. Bacteria which have not been previously identified in pneumonia and which were identified in this study only in pneumonia patients are shown in red. Bacteria which have not been previously identified in pneumonia patients and which were identified in this study in both pneumonia and control patients are shown in blue, whereas bacteria which have been identified in this study only in control subjects and not in pneumonia patients are shown in green.

Molecular assays were positive for at least one bacterium for 129 out of 185 bronchoalveolar lavage (BAL) samples from patients with pneumonia as well as from 13 out of 25 from control individuals (p = 0.07). Bacterial clone libraries from amplified 16S rDNA genes (nearly 4,000 clones that contained exploitable sequences were included) identified 157 different bacteria at the species level. Detailed data about the relative abundance and richness of each species in their corresponding library are summarized in data S1 and S2 in supplementary informations. Bacterial clone libraries of patients showed that 44 libraries were characterized by the presence of only one bacterium, 40 libraries were polybacterial but dominated by one bacterium (50% of the clones in the library), whereas 45 libraries were polybacterial without any dominant bacterium (Fig. 1). Bacterial clone libraries of controls showed that 2 libraries were characterized by the presence of only one bacterium, 4 libraries were polybacterial but dominated by one bacterium (50% of the clones in the library), whereas 7 libraries were polybacterial but without any dominant bacterium (Fig. 1). Patients exhibited up to 15 bacteria in their BAL fluids (mean ± SD; 3.48±2.80) (Tables 1 and 2). Overall, patients exhibited 146 different species belonging to 7 different phyla (13 classes, 23 orders, 44 families and 71 genera) of which 73 had not been previously observed in BAL from pneumonia, whereas bacterial clone libraries of controls identified 38 species belonging to 4 different phyla (9 classes, 13 orders, 22 families and 27 genera). In patients, aerobic gram-negative bacilli, gram-positive cocci, and anaerobic bacteria from oropharyngeal flora were the most frequent bacteria identified (Tables S1,S2,S3,S4, Fig. 2). Surprisingly, bacteria that are usually associated with other diseases such as the gram-positive anaerobe *Atopobium vaginae*, or from unexpected animal origins, such as *Enterococcus canintestini*, were alsoc found. Moreover, 51 strictly anaerobic bacteria (35%) were found in patients versus 17 anaerobic bacteria (44%) found in controls (p = 0.26). Among those bacteria which were identified in controls, 24 bacteria were also identified in patients (Fig. S1, Tables S2,S3,S4), including *Pseudomonas aeruginosa* sequences respectively identified in 100% and 86% from 2 different clones libraries from 2 immunocompromised controls. In the second clone library, the 14% of the remaining sequences included *Achromobacter xylosoxidans*, which also is a typical bacterium of nosocomial pneumonia. *Stenotrophomonas maltophilia* sequences were found in 10% of clone library of another immunocompromised control, along with 5 other bacteria. Similarly, sequences of *Streptococcus mitis* (7% of the clone library) was identified along with 12 other bacteria in a control with acute respiratory distress syndrome (ARDS) and a history of aspiration pneumonia (AP) 6 days before BAL sampling. Additionally, *Arcobacter cryaerophilus, Atopobium parvulum*, *Lachnospiraceae bacterium, Prevotella melaninogenica*, and *Prevotella pallens* were significantly more frequent in controls than in patients (p = 0.01, 0.01, 0.001, 0.01 and 0.01 respectively) (Table S4).

**Table 1:** Summary of the number of bacteria, fungi and viruses identified by molecular assays in BAL fluids.

	*Pneumonia cohorts*				*Pneumonia patients (n = 185)*	*CS (n = 25)*
	CAP (n = 32)	VAP (n = 106)	NV ICU-P (n = 22)	AP (n = 25)		
N° Bacteria/Sample						
10–15	0	3	0	1	4	1
5–9	5	18	5	5	33	4
4	1	10	2	1	14	4
3	3	13	3	3	22	0
2	3	6	1	2	12	2
1	10	27	5	2	44	2
0	10	29	6	11	56	12
N° species/positive sample ± SD	2.68±2.07	3.49±2.87	3.43±2.22	4.78±3.68	3.48±2.80	4.61±2.95
N° Fungi/Sample						
5	1	0	0	0	1	0
3	0	0	1	0	1	0
2	1	2	3	1	7	1
1	2	16	2	3	23	5
0	28	88	16	22	154	19
N° species/positive sample ± SD	2.25±1.89	1.11±0.32	1.83±0.75	1.25±0.5	1.40±0.83	1.16±0.40
N° Viruses/Sample						
2	3	6	3	0	12	0
1	7	47	8	5	67	11
0	22	53	11	20	106	14
N° species/positive sample ± SD	1.3±0.48	1.11±0.31	1.27±0.46	1±0	1.15±0.36	1.09±0.30

CAP, community-associated pneumonia; VAP, ventilator-associated pneumonia; NV ICU-P, non-ventilator ICU pneumonia; AP, aspiration pneumonia; CS, control subjects.

**Table 2:** Microbiota composition of positive BAL fluids by molecular assays.

	Pneumonia cohorts				Pneumonia patients (n = 185)	CS (n = 25)	P value
	CAP (n = 32)	VAP (n = 106)	NV ICU-P (n = 22)	AP (n = 25)			
Only bacteria	12	34	5	11	62	6	0.34
Only fungi	0	2	1	1	4	1	0.57
Only viruses	3	9	3	2	17	3	0.65
Bacteria and fungi	3	4	3	1	11	2	0.68
Bacteria and viruses	6	33	6	2	47	4	0.30
Fungi and viruses	0	4	0	1	5	3	**0.02**
Bacteria and fungi and viruses	1	7	2	0	10	1	0.76
Total positive samples	25	93	20	18	156	20	0.58

CAP, community-associated pneumonia; VAP, ventilator-associated pneumonia; NV ICU-P, non-ventilator ICU pneumonia; AP, aspiration pneumonia; CS, control subjects.

**Figure 3:**
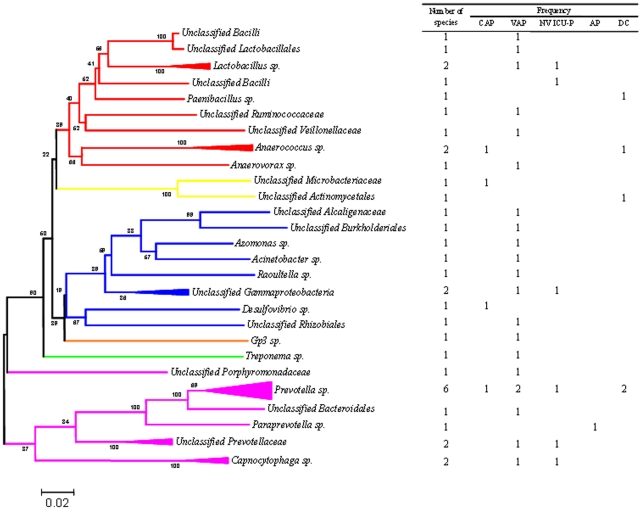
A phylogenetic tree inferred from 16S rDNA sequences of novel bacterial phylotypes. These novel phylotypes exhibited sequence similarities of less than 98% to known bacteria available in the GenBank database, and they were classified *in silico* using “Classifier” program. Phylotypes are reported according to their genus or by the last possible classification determined by the program. When possible, phylotypes with the same classification are clustered together. The frequency of phylotypes in each cohort is shown on the right.. *Bacteroidetes* are shown in purple, *Firmicutes* in red, *Proteobacteria* in blue, *Actinobacteria* in yellow, *Acidobacteria* in orange and *Spirochaetes* in green. CAP, community-acquired pneumonia; VAP, ventilator-associated pneumonia; NV ICU-P, non-ventilator ICU pneumonia; AP, aspiration pneumonia; and CS, control subjects.

Bacterial clone libraries surprisingly showed 37 new phylotypes with 16S rDNA sequence similarity lower than 98% to known bacteria available in the GenBank database (Data S1 and S2). Among them, 32 novel bacterial phylotypes were identified in BAL from patients, whereas 5 novel phylotypes were identified in BAL from controls (Fig. 3). Novel bacterial phylotypes identified in patients were more diversified, as they belonged to 6 different phyla including *Bacteroidetes* (11 phylotypes), *Firmicutes* (9 phylotypes), *Proteobacteria* (9 phylotypes), *Actinobacteria* (one phylotypes), *Acidobacteria* (one phylotype) and *Spirochaetes* (one phylotype). Novel species identified in controls belong to *Bacteroidetes* (2 phylotypes), *Firmicutes* (2 phylotypes) and *Actinobacteria* (one phylotype). *Prevotellaceae* phylotypes represent 24% of all novel phylotypes identified and they were exhibited in patients with pneumonia as well as in control subjects (Fig. 3). Results obtained using routine BAL and blood culture are available Text S1.

### Fungal microbiota as evaluated by the intergenic spacer of 18S rDNA

At least one fungus was found in 31 BAL patient samples and in 6 from controls (p = 0.37). Positive patients exhibit up to 5 fungi in their BAL fluids (mean ± SD; 1.40±0.83) (Tables 1 and 2). Detailed data about the relative abundance and richness of each species in their corresponding library are also summarized in summary files (Data S1 and S2) in supplementary informations. Fungal microbiota obtained from patients showed the presence of 22 different species belonging to 2 phyla (8 orders, 11 families and 12 genera) among which 6 phylotypes had not been previously identified in BAL fluids from pneumonia. Clone libraries from controls, identified 5 fungi belonging to one phylum (2 orders, 4 families and 3 genera) among which 3 fungi were also identified in patients. *Candida* species were the most common fungal species identified (Tables S1,S2,S3 and S5). Environmental fungi, which usually colonize water, food debris and humid building surfaces, were more notably identified in our study than in previous pneumonia studies. Furthermore, tree fungi belonging to *Basidiomycota* phylum, *Sporidiobolales* sp., *Cryptococcus victoriae* and *Hyphoderma praetermissum*, were found for the first time in pneumonia BAL samples in the present study, while *Candida utilis* and *Periconia macrospinosa* were identified only in controls. Additionally, *Candida utilis* was significantly more frequent in controls than in patients (p = 0.01). Results of fungi obtained using a routine BAL culture are available in Text S1.

### Viruses and fastidious bacterial pathogens detected by qPCR

Four pneumonia patients were found to be positive for fastidious bacteria *Chlamydia pscitacii* (1 case), *Mycobacterium* sp. (2 cases) and *Mycoplasma pneumoniae* (2 cases) by qPCR. In addition, qPCR enabled the detection of 7 different viruses. Quantitative data of microorganisms identified by qPCR (loads or Cycle threshold) are also provided and summarized in supplementary information (Data S1 and S2). Our study showed that at least one virus was identified in 74 BAL samples from patients, and 11 from controls (p = 0.95). HSV and CMV were the most commonly identified viruses. While the prevalence of these two viruses in patients was not significantly different from that of controls (Table S6), CMV was more frequently identified in pneumonia patients than in controls. HSV and CMV coinfection was found in BAL samples from 5 VAP patients, 2 community-acquired pneumonia (CAP) patients and 2 non-ventilator ICU pneumonia (NV ICU-P) patients and one control subject. Coinfection with CMV and respiratory syncytial virus type A was detected in a BAL from one NV-ICU-P patient, and both HSV and VZV were identified in a BAL from a CAP patient. Rhinovirus was identified in a control with ARDS, urinary infection and sinusitis. Parainfluenza virus-1 was detected in 3 VAP patients and an immunocompetent control with a pulmonary contusion. Results obtained using routine serology and antigenemia for viruses and fastidious pathogens are available in Text S1

### Comparison of the microbiota between pneumonias and controls

**Figure 4:**
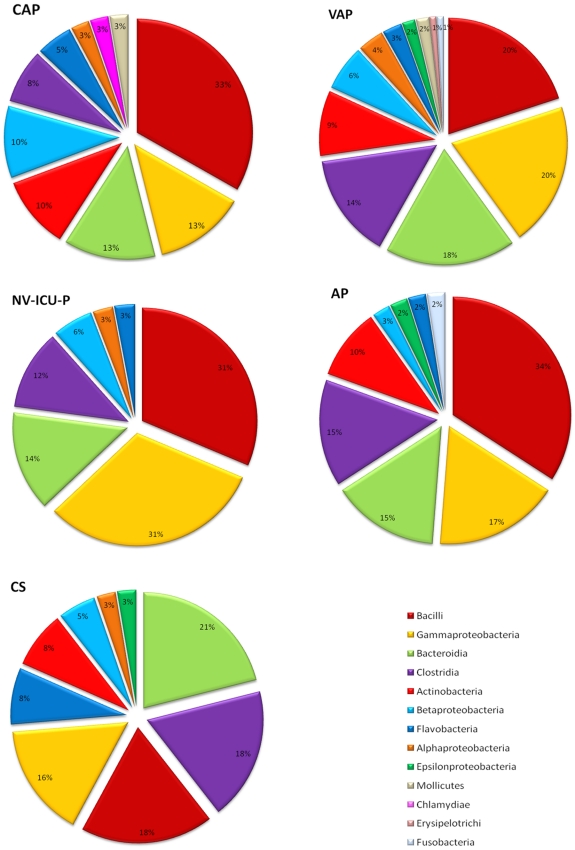
Differences in bacterial microbiota composition between community- acquired pneumonia (CAP), ventilator-associated pneumonia (VAP), non-ventilator ICU pneumonia (NV ICU-P), aspiration pneumonia (AP) and control subjects (CS) cohorts. Bacteria are shown according to their classes. Distribution of bacterial classes in each cohort is expressed as a percentage. Bacterial classes and their corresponding colours are indicated in the bottom right corner.

**Figure 5:**
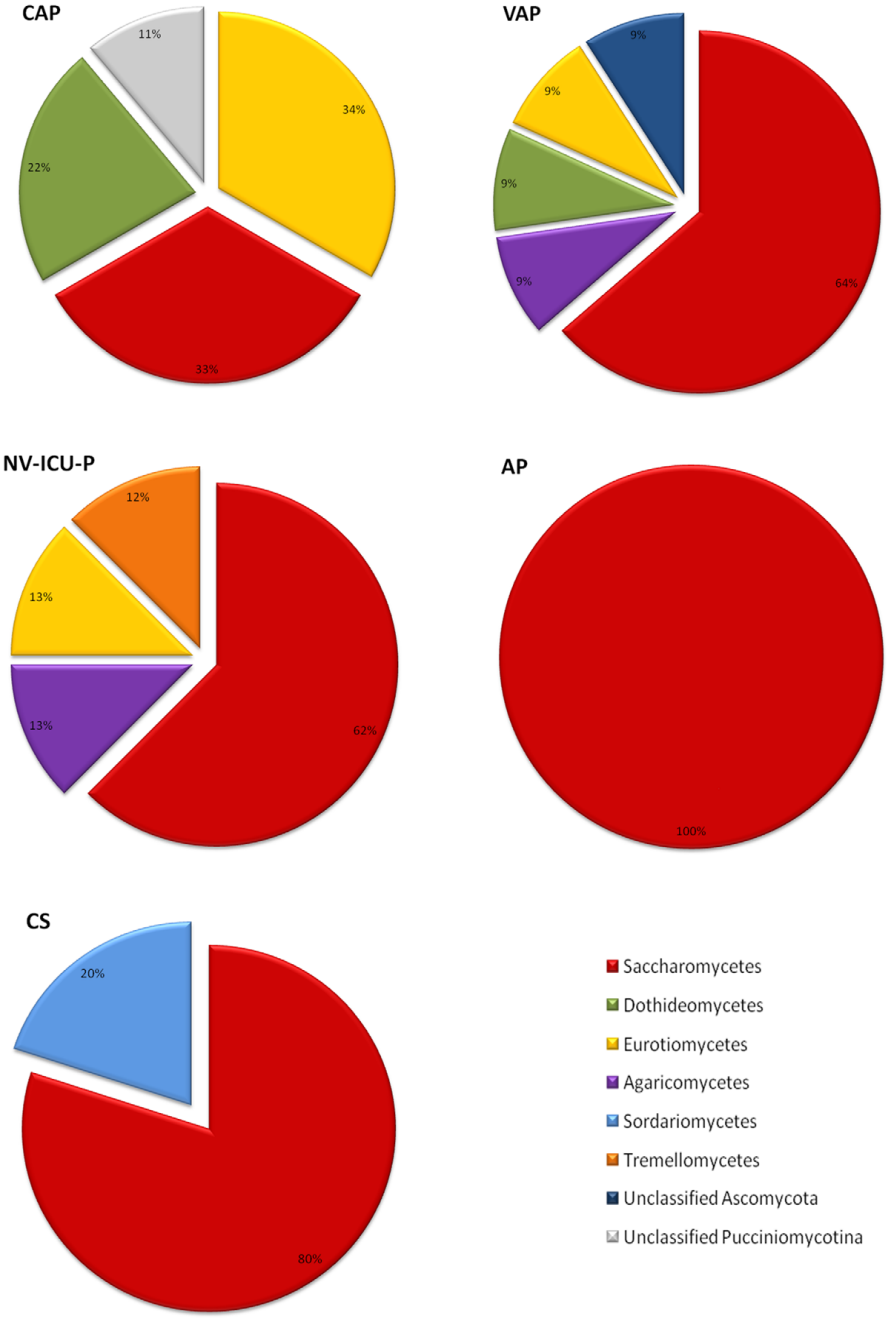
Differences in fungal microbiota composition between community-associated pneumonia (CAP), ventilator-associated pneumonia (VAP), non-ventilator ICU pneumonia (NV ICU-P), aspiration pneumonia (AP) and control subjects (CS) cohorts. Fungi are presented according to their classes. Distribution of fungal classes in each cohort is expressed as a percentage. Fungal classes and their corresponding colours are indicated in the bottom right corner.

Overall, bacterial difference between patients and controls showed that bacteria belonging to *Bacilli* and *Gammaproteobacteria* were dominant in patients, whereas anaerobic bacteria related to *Bacteroidia* (represented essentially by *Prevotella* species) and *Clostridia* were dominant in controls (Fig. 4) (p<0.01). *Mollicutes*, which are represented by the *Mycoplasma* genus, were only detected in patients with CAP and VAP (Fig. 4). As for fungal species, members of *Saccharomycetes* were ubiquitously identified in all cohorts. *Eurotiomycetes*, which are represented by *Aspergillus*, *Penicillium* and *Cladophialophora* genera, were dominant in the CAP cohort (Fig. 5). *Tremellomycetes*, represented by the *Cryptococcus* genus, was only identified in the NV-ICU-P cohort, whereas *Agaricomycetes* and an unclassified *Ascomycota* (Melanized limestone *ascomycetes*) were only identified in the VAP cohort. In addition, *Sordariomycetes*, which is represented by the *Periconia* genus, was only identified in controls.

At the specie-level, 58 bacteria, 7 fungi and 5 viruses were common to at least two cohorts, among which *Pseudomonas aeruginosa*, *Streptococcus mitis*, *Prevotella melaninogenica*, *Peptostreptococcus stomatis*, *Candida albicans* and HSV were commonly identified irrespective of cohorts, whereas *Haemophilus influenzae, Staphylococcus aureus*, *Streptococcus* genomosp. C4, *Streptococcus parasanguinis* and *Streptococcus pneumoniae* were commonly identified in patients regardless of pneumonia type (Fig. S2). Additionally, 24 bacteria, 3 fungi and 3 viruses were common to controls and at least one pneumonia cohort, whereas 34 bacteria, 4 fungi and 2 viruses were common to at least two pneumonia cohorts (Fig. S2). In contrast, many microorganisms (102 bacteria, 17 fungi and 2 viruses) were restricted to one of cohorts (17 bacteria and 5 fungi only were identified in the CAP cohort; 54 bacteria and 6 fungi only were identified in the VAP cohort; 8 bacteria, 4 fungi and one virus only were identified in the NV ICU-P cohort; 9 bacteria only were identified in the AP cohort; 14 bacteria, 2 fungi and one virus only were identified in controls) (Fig. S2).

### Abundance and specie richness in the libraries and correlation with clinical data

Microbial profiles of positive pneumonia BAL fluids showed that 40 (25%) were characterized by the presence of one microorganism, whereas 116 (75%) were polymicrobial. In controls, 4 (20%) of BAL fluids were characterized by the presence of one microorganism, whereas 16 (80%) were polymicrobial (Data S1 and S2). Available clinical data for patients and controls showed that monobacterial patients were more frequently, but statistically insignificant, subjected to initial antibiotic therapy than polymicrobial ones (p = 0.12; Table 3 and Table S7). In ventilated subjects, monomicrobial patients have a slightly shorter period of mechanical ventilation prior to the pneumonia episode as compared to polymicrobials. Monomicrobial controls have a remarkably shorter period of mechanical ventilation before sampling compared to polymicrobials (p = 0.25; Table 3). The same observation was showed for length of ICU stay prior to sampling and for total length of hospital stay. According to these observations, the polymicrobial profiles of controls seem to be partially related to the high duration of ICU stay before sampling. However, the ICU mortality was higher in monomicrobial patients than in polymicrobial ones (p = 0.02). The ICU mortality rate was higher in pneumonia patients for whom BAL fluids exhibited only viruses or fungi, or both than in monobacterial or polybacterial patients (p = 0.01; Table S7). A higher but not statistically significant ICU mortality was also observed in pneumonia patients for whom BAL fluids exhibited only viruses or fungi, or both than in controls with the same profile (p = 0.07; Table S7).

**Table 3:** Clinical data in monomicrobial and polymicrobial episodes.

	*Pneumonia patients (n = 135/185)*		*Control subjects (n = 22/25)*	
	Monomicrobial	Polymicrobial	Monomicrobial	Polymicrobial
Case number	32	82	3	15
Age, yr (SD)	58.5 (13.9)	62.9 (14.7)	74 (13.4)	54.2 (17.3)
Male gender	16	46	3	7
Female gender	16	36	0	8
Immunocompromized	15	20	1	4
ARDS	15	19	0	4
CPIS (SD)	3.7 (1.8)	3.6 (1.7)	4.6 (0.5)	4 (1.4)
SOFA score (SD)	6.9 (3.1)	6.7 (3.6)	6.3 (3.7)	5.4 (2.6)
Radiologic score (SD)	5.5 (2.9)	4.8 (3.2)	3.6 (3.2)	5.3 (2.8)
Temperature, °C (SD)	37.6 (1)	37.8 (1.7)	37.2 (1.2)	37.6 (1.4)
Initial antibiotic therapy	18	33	2	9
Less than 2 days prior to sampling	6	17	0	3
3 days or more prior to sampling	12	16	2	6
Length of ICU stay prior to sampling, d (SD)	6.1 (9.1)	6.3 (10)	1.6 (1.1)	13.1 (15.6)
Total length of hospital stay, d (SD)	26 (29.7)	28.2 (23.3)	4.3 (1.5)	36.6 (35.9)
Length of MV prior to sampling, d (SD)	6.8 (10.3)	7.1 (11.2)	1.3 (0.5)	6.3 (7.2)
Sepsis	7	19	0	3
Septic shock	15	38	1	7
ICU mortality (%)	16 (50)	23 (28)	1 (33)	3 (20)

### Comparison of lung microbiota between different studies

We next compared the bacterial communities found in our study to those found in lung specimens in five previous studies which were based on 16S rDNA amplification [9], [11], [14]–[16]. Comparative analyses of lung microbiota between these studies showed that 147 different genera were found in all of them. Among these genera, 70 genera are widely distributed within the studies including *Gemella*, *Haemophilus*, *Megasphaera*, *Neisseria*, *Porphyromonas*, *Prevotella*, *Pseudomonas*, *Staphylococcus* and *Streptococcus* genera which have commonly been found irrespective of study. In contrast, 77 genera were restrictively identified across the studies (Table 4). However, at the species level (only the studies that determined bacterial species were included [9], [11], [14]), comparative analyses showed that from 59 bacteria commonly distributed within the studies, *Escherichia coli*, *Haemophilus influenzae*, *Prevotella oris*, *Pseudomonas aeruginosa*, *Staphylococcus aureus* and *Streptococcus mitis* were commonly found in the four studies. In contrast, 291 bacteria were restrictively identified across the studies (Fig. S3). Consequently, comparative analysis at the specie-level showed that some bacteria, such as *Pseudomonas aeruginosa* and Staphylococci, are commonly found in pulmonary specimens. However, the pattern of distribution of many other species is distinctly heterogeneous and depending on the specific study and disease. Variation of lung microbiota, from one individual to another and from one study to another, suggests that the repertoire of microorganisms associated with respiratory infections still remains incompletely understood.

**Table 4:** Comparison of lung microbiota between different studies.

	*Lung microbiome studies (only lung samples)*					
	Our study	Harris et al. [9]	Bittar et al. [11]	Bahrani-mougeot et al. [14]	Erb-Downward et al. [15]	Hilty et al. [16]
Studied diseases	ICU pneumonia	CF	CF	VAP	COPD	Asthma
N° of studied individuals	210 (140 positive cases)	57 (42 positive cases)	29	39 (16 positive cases)	20 (14 BAL; 6 explants)	24
N° of cases	185 (129 positive cases)	36 (28 positive cases)	25	39 (16 positive cases)	10 (4 BAL; 6 explants)	11
N° of controls	25 (13 positive cases)	21 (14 positive cases)	4	No	10 (BAL)	13
Profile of controls	Without pulmonary infections	With pulmonary infections	With bronchiectasis	No	Smokers and non smokers healthy peoples	COPD and healthy controls
Type of samples	BAL	BAL	Sputa	BAL	BAL and lung explants	Left upper lobe brush
N° of determined species	160	121	57	53	ND	20
Total N° of genera	81*	56	34	28	74	39
N° of genera common to other studies	60	42	29	24	43	37
N° of genera restrictive to each study	21	14	5	4	31	2
N° genera/patients	76	37	12	28	51	31
N° genera/controls	28	41	31	No	46	30
N° genera common to Pts and Cs	21	22	9	No	21	20
Mean genera/patient ± SD	2.88±2.20	ND	5±2.41	ND	ND	ND
Mean genera/control ± SD	3.84±2.26	ND	5.75±0.95	ND	ND	ND

CF; Cystic fibrosis, COPD; Chronic obstructive pulmonary disease, ND; not determined,

* ; included novel phylotypes successfully classified at the genus level.

## Discussion

Previous studies performed on respiratory specimens showed that unexpected bacteria are increasingly identified, as well as studies describing isolated cases of respiratory infection due to an unexpected bacterium that was detected using molecular techniques [9], [11], [14], [17]–[20]. This study extends the analyses to bacteria, fungi and viruses in a large population of ICU pneumonia using comprehensive molecular testing. Our results demonstrate that nearly 50% of the microbial species found had not been previously reported in lung samples from pneumonia. Therefore, the composition of ICU-pneumonia microbiota is more complex, more extensive and more diverse than originally expected.

However, we raise the question on the actual role of these microorganisms in pneumonia. Indeed, our study reveals that some pathogens that till now had been considered typical for ICU pneumonia, such as *Pseudomonas aeruginosa* and *Streptococcus* species, or viruses, such CMV and HSV, can be detected as commonly in controls as in patients (Fig. S1 and S2). This result is emphasized by more recent studies by Erb-Downward et *al*. and Hilty et *al.* who showed that a community of lung-resident bacteria including *Pseudomonas* and *Streptococcus* genera can be identified in patients with chronic obstructive disease or asthma, as well as in healthy people [15], [16]. Our study agrees with the recent literature and highlights the existence of a core pulmonary microbiota, confirming the non-sterility of the lung [15], [16].

More interestingly, we showed that pulmonary microbiota heterogeneity can be observed between patients and controls, among pneumonia cohorts and among patients within the same cohort. High pulmonary microbiota heterogeneity was also observed between our study and other previous works performed on cystic fibrosis or VAP [9], [11], [14] (Fig. S3). We found that some bacteria were commonly identified in all studies, whereas many others were only identified in one study, and most of these were unexpected. Consequently, lung microbiota can vary greatly between individuals, depending on underlying diseases, habits and geographic origin. Additionally, these unexpected microorganisms may explain a lack of response to drug therapies in some pneumonia patients. Therefore, the possible extension of empiric treatments to cover a large spectrum of microorganisms, especially for patients who do not respond adequately to initial treatment, is questionable.

Another interesting observation was that mixed infection was observed in many BAL fluids from pneumonia patients. Interestingly, recent works report that probable interactions between parasitic species can occur in their host, and these reports also show that infection with a given microorganism may increase or decrease susceptibility to infection by another one or can create a cross-immunity response [21]–[27]. Such interaction remains to be investigated.

Moreover, by comparing molecular testing to standard routine methods, this study reveals that many pneumonia-associated pathogens are fastidious or uncultured and highlights a wide discrepancy between culture and molecular microorganism repertoire. Our study also shows that the molecular assay remains a more efficient method to detect microorganisms in the pneumonia samples, independently of atmospheric conditions and medium nutrient supplements, which are particularly important for culture, especially for fastidious microorganisms. In addition, microorganism diagnosis was obtained for 156 (84%) episodes of pneumonia by molecular tools compared with 120 (65%) pneumonia episodes for which microorganism diagnosis was successfully done by culture (Table S8). In particular, molecular tools seem to be far more sensitive than culture for bacterial detection. This observation is based on the high number of microorganisms, especially bacteria which were identified by molecular methods compared with those detected by culture. In fact, standard and special BAL cultures identified few, essentially easily-grown and strictly aerobic or facultative anaerobic bacteria (23 species) compared to molecular tools which identified 160 bacterial species (p<0.001) (Fig S4 and S5). Molecular tools enabled the identification of unexpected bacteria which usually colonize vaginal tracts, such as *Atopobium vaginae* and *Peptoniphilus lacrimalis*, or of other bacteria coming from unexpected animal origins, such as *Chlamydia pscittasi, Enterococcus canintestini* and *Streptococcus bovis*, or of potentially known to be associated with other diseases, such as *Tropheryma whipplei*, which were not identified by culture. Furthermore molecular tools allowed the detection of pathogenic bacteria such as *Mycobacterium* sp. and *Mycoplasma pneumoneae*, for which identification attempts by culture using specific media were failed due to culture biases. Moreover, all bacteria that were first associated with pneumonia in the present study were exclusively identified by molecular methods. These findings are coherent to results from previous studies on bacterial communities of respiratory diseases, including pneumonia, which showed that molecular assays are more sensitive than culture [9], [11], [14]. However, although molecular approaches identified more fungal species than culture, fungal diagnoses were positive for 79 (42%) episodes of pneumonia by culture compared with 31 (17%) pneumonia episodes for which fungal diagnosis was successfully obtained using molecular tools. Thus, fungal BAL culture was more sensitive to detect some cultured fungi, such as *Candida* species, than molecular approaches.

Another important finding was the high number of novel bacterial species never previously described to date (bacteria with BLASTn similarity less than 98%). This result is concordant with similar studies of pneumonia and cystic fibrosis subjects [9], [14] and shows that in respiratory infections, more complex bacteria populations can exist, among which novel bacteria had never been previously identified. Moreover, this finding was also supported by other studies performed on endodontic infections, demonstrating that many novel bacteria essentially resident in the oropharyngeal and dental plaque flora can be detected in these infections [28], [29]. The oropharyngeal and dental plaque flora is potentially suspected to be a reservoir and, thus, the source of ICU pneumonia pathogens, which could suggest that these novel bacteria were inhaled through oropharyngeal tracts [30], [31].

Nevertheless, molecular tools alone cannot give positive results in some cases, or they could just identify microorganisms known to be commensal or less pathogenic, where it may be useful to perform other tests, such as serology. This was the situation for 5 pneumonia patients for whom serology provided evidence for influenza A virus infection, whereas qPCR performed on their BAL fluids was negative. Moreover, by combining culture-based methods, blood culture and serology to molecular approaches we significantly increase the probability to detect microorganisms in the pneumonia episodes. In fact, by using these exhaustive laboratory diagnostic tools, we failed to identify a microbial agent in only 7% of the pneumonia episodes, which is significant when compared to previous studies where the microbial agent was not found in more than 30% of episodes of pneumonia (p<0.001) [32]. However, the clinical significance of these microorganisms and their role in the etiology of pneumonia remain difficult to be cleared as their correlation with the disease causation remains to be studied and confirmed in the future. Nevertheless, our findings suggest that it would be highly recommended to develop a rapid molecular test to target, besides typical pathogens, potential pathogens known to be fastidious or uncultured (such as anaerobic ones), and that it would be useful to add it to existing routine standard techniques.

In summary, our study reveals that the respiratory microbiota is more complex than expected.

## Materials and Methods

### Patients and clinical samples

A large study was implemented in our laboratory over a three-year period (January 2007 through December 2009) to perform an exhaustive etiologic diagnosis of pneumonia. The study involved three ICUs in the public hospitals of Marseille, France (One medical ICU and two medico-surgical ICUs). A total of 185 BAL fluids, 185 blood samples and 185 urinary samples from 130 ICU pneumonia patients were studied. A diagnosis of community-acquired pneumonia, ventilator-associated pneumonia and aspiration pneumonia was defined as previously described [32]–[34]. BAL and blood sampling were performed as previously described [35]. A cohort of 25 ICU patients without pneumonia was studied as controls. Pneumonia patients exhibited 32 episodes of community-associated pneumonia, 106 episodes of ventilator-associated pneumonia, 22 episodes of non-ventilator ICU pneumonia and 25 episodes of aspiration pneumonia. Further clinical data are summarized in SI Table S9. Written informed consent was obtained from patients' family members. The project was approved by the Local Ethics Committee (comité d'éthique de l'institut fédératif de recherché de la Faculté de Médecine de Marseille (IFR48, Marseille, France), and the permit number was 07–026.

### Nucleic acid extraction, PCR amplification, cloning and sequencing

Bacterial and fungal DNA extraction from BAL samples was performed on a MagnaPure LC workstation (Roche Diagnostics, Meylan, France), using a MagNa Pure LC DNA Isolation Kit II (Roche Diagnostics) as previously described [12]. Viral nucleic acids were extracted from 200 µL of BAL fluids using an MDX workstation and the QIAamp Virus BioRobot MDx Kit according to the manufacturer's instructions. DNA was tested by PCR for bacteria using broad-range primers targeting the 16S rDNA gene; PCR was also used to test for universal fungi using broad-range primers targeting intergenic spacer of 18S rDNA gene (Eurogentec, Seraing, Belgium) (Table 5). PCR product was cloned and approximately 48 clones were screened per library. PCR, cloning and sequencing were performed as previously described [12]. The obtained sequences were assembled and analyzed by chromaspro software and then BLASTed against those available in the GenBank database (www.ncbi.nlm.nih.gov) for species identification. Chimeric sequence search was performed with Black Box Chimera Check (B2C2) program [36] and by examining the BLAST profile of each sequence. Suspected chimeric sequences were discarded from the study. Sequences showing a similarity of >98% were considered to be known species, whereas sequences showing a similarity of <98% were considered to be novel species. *Legionella* sp., *Afipia* sp.,*Bradyrhizobium* sp., *Azorhizobium* sp., *Mesorhizobium* sp., *Balneatrix alpaca* and *Pneumocystis carinii* were tested by PCR using specific primers followed by sequencing of PCR products (Table 5). The sequences have been deposited in the GenBank database (accession N° JF893554–JF893750).

**Table 5:** Primers and probes used in molecular assays.

*Microorganisms*	*Gene*	*Forward primer*	*Reverse primer*	*Probe*
Bacteria				
Universal bacteria	16 s rRNA	5′-CAGCAGCCGCGGTAATAC-3′	5′-ACGGCTACCTTG TTACGACTT-3′	Cloning and sequencing
*Legionella* sp.	mip	5′-GGGRATTVTTTATGAAGAAGARAYTGG-3′	5′-TCRTTNGGDCCDATNGGNCCDCC-3′	Sequencing
*Mycobacterium sp.*	ITS	5′-GGGTGGGGTGTGGTGTTTGA-3′	5′-CAAGGCATCCACCATGCGC-3′	5′-TGGATAGTGGTTGCGAGCATC-3′
*M. tuberculosis*				5′-GCTAGCCGGCAGCGTATCCAT-3′
*M. avium* group				5′-GGCCGGCGTTCATCGAAAT-3′
*Afipia* sp.	rpoB	5′-TGAAGATGGTCAAGGTCTTCG T-3′	5′-GTC CGACTTSA HGTCAGCAT-3′	Sequencing
*Bosea* sp.				
*Bradyrhizobium* sp.				
*Parachlamydia* sp.	ADP	5′-TAG TGATCTGCTACGGGATTT-3′	5′-TTG GATTAGGATATTGCAATT T-3′	5′-AACCTTGTAGAAGTAACCTGGAAGAACCAGC-3′
*Azorhizobium* sp.	nodA	5′-TGCATAATTACTGTGCACCAGAG-3′	5′-TCAACACCCTCTGGCCAACG-3′	Sequencing
*Mesorhizobium* sp.	16 s rRNA	5′-GGAGCTAATACCGTATACGTC-3′	5′-CAAGTAAACTTGCCAACGGCTAG-3′	Sequencing
*B. alpica*	Cpn60	5′-CGCTGCAGCGGTAGTTGAGC-3′	5′-TAAGGACTATCCAGCTCAGCAGACATAT-3′	Sequencing
*C. burnetii*	IS1111	5′-CAAGAAACGTATCGCTGTGGC-3′	5′-CACAGAGCCACCGTATGAATC-3′	5′- CCGAGTTCGAAACAATGAGGGCTG-3′
*C. pneumoniae*	omp2	5′-GATTCGTCGCTAGTGCGGA-3′	5′-GTCTAACCTTCTTCGCTGTCA-3′	5′-ACAAAGCCAGCACCTGTTCCT-3′
*C. psittaci*	omp2	5′-TTCTGTGATAAAGAATTTTATCCT-3′	5′-CGGACACAATACATTTTGCCG-3′	5′-CCAGTGCCAACCAGTAGACGCTA-3′
*M. pneumoniae*	Enolase	5′-ATGTCCCGCTCCGAACGAA-3′	5′-CCAATTCCAGTTCAATTTGCAA-3′	5′-TGCCAAATACAATCGC-3′
Fungi				
Universel fungi	ITS	5′-TCCGTAGGTGAACCTGCGG-3′	5′-GCTGCGTTCTTCATCGATGC-3′	Cloning and sequencing
*Aspergillus* sp.	28S rDNA	5′-CTCGGAATGTATCACCTCTCGG-3′	5′-TCCTCGGTCCAGGCAGG-3′	5′-GTCTTATAGCCGAGGGTGCAATGCG-3′-
*Pneumocystis carinii*	DHFR	5′-GTTGCACTTACAACTTCTTATGG-3′	5′-TAGATCCAGAGATTCATTTCGAG-3′	Sequencing
Viruses				
Mimivirus	Capside	5′-GATAAACATTATGGTGACTG-3′	5′-AGGAACATACAGAGTATATG-3′	5′-ATCATGAAAAGGGTCTTGCTA-3′
RSV A	Gene n	5′-AGATCAACTTCTGTCATCCAGCAA-3′	5′-GCACATCATATTTAGGAGTATCAAT-3′	5′-CTTTGCCATACTCAATGAACAAAC-3′
RSV B	Gene n	5′-AAG TGCAAATGATAAATTCACAGGA-3′	5′-TAGTATCCAGCATCTTTAAGTZTCTTTATAG-3′	5′-CACCATCCAACGGAGCAGAGGAGAT-3′
Influenza A	Gene m	5′-GGACTGCAGCGTAGACGCTT-3′	5′-CATYCTGTTGTATATGAGGCCCAT-3′	5′-CTCAGTTATTCTGCTGGTGCACTTGCCA-3′
Influenza B	Gene h	5′-AATACGGTGGATTAAATAGCAA-3′	5′-CCAGCAATAGCTCCGAAGAAA-3′	5′-CACCCATATTGGGCAATTTCCTATGGC-3′
Parainfluenza 1	Hg/Ne	5′-CATTATCAATTGGTGATGC-3′	5′-CTTAAATTCAGATATGTATCCTG-3′	5′-CTTAATCACTCAAGGATGTGCAGATATA-3′
Parainfluenza 3	Hg/Ne	5′-CTCGAGGTTGTCAGGATATAG-3′	5′-CTTGGGAGTTGAACACAGTT-3′	5′-AATAACTGTAAACTCAGACTTGGTACCTGACTT-3′
Rhinovirus	5′ NCR	5′-GCACTTCTGTTTCCCC-3′	5′-GGCAGCCACGCAGGCT-3′	5′-AGCCTCATCTGCCAGGTCTA-3′
Metapneumovirus	Gene N	5′-AACCGTGTACTAAGTGATGCACTC-3′	5′-CATTGTTTGACCGGCCCCATAA-3′	5′-CTTTGCCATACTCAATGAACAAAC-3′
Enterovirus	5′NC	5′-CCCTGAATGCGGCTAATCC-3′	5′-ATTGTCACCATAAGCAGCCA-3′	5′-CADGGACACCCAAAGTAGTCGGTTCC-3′
Coronavirus OC-43	Pol	5′-CGCCGCCTTATTAAAGATGTTG-3′	5′-GGCATAGCACGATCACACTTAGG-3′	5′-AATCCTGTACTTATGGGTTGGGATT-3′
Coronavirus 229-E	Pol	5′-TGGAGCGAGGATCGTGTTC-3′	5′-TAGGCTGTGACAGCTTTTGCA-3′	5′-TGTTCTCACGCTGCTGTTGATTCGCT-3′
Coronavirus NL-63	Replicase	5′-TGTTGTAGTAGGTGGTTGTGTAACATCT-3′	5′-AATTTTTGTGCACCAGTATCAAGTTT-3′	5′ATGTTTCACCAATTGTTAGTGAGAAAATTTCTGTTATGGA-3′
HSV	Pol	5′-CATCACCGACCCGGAGAGGGAC-3′	5′-GGGCCAGGCGCTTGTTGGTGTA-3′	5′-CCGCCGAACTGAGCAGACACCCGCGC-3′
VZV	Pol	5′-GGTTAAACGTTTGAATCCATCC-3′	5′-CAGCAGACTTTCTCGAACGT-3′	5′-ATGCCACCTTTACAGTTGGAGGAA-3′
CMV	pp65	5′-GCAGCCACGGGATCGTACT-3′	5′-GGCTTTTACCTCACACGAGCATT-3′	5′-CGCGAGACCGTGGAACTGCG-3′

### BAL and blood cultures and phenotypic identifications

Standard bacteriological BAL culture and blood culture as phenotypic identification of isolated bacteria were performed as previously described [11], [37]. A 10^4^ CFU cut-off defined a positive BAL culture. Blood culture were processed as previously described [37]. Identification of fungi present in BAL or blood samples was performed using a standard culture as previously described [38], [39]. Viral culture for Cytomegalovirus, herpes simplex virus, parainfluenza viruses (types 1 and 3), respiratory syncytial virus, varicella-zoster virus, influenza viruses (type A and B), and enterovirus was performed using shell-vial culture as previously described [4], [40]. Amoeba co-culture were performed in microplates on *Acanthamoeba polyphaga* as previously described [41]. Tentative isolations of *Mycobacterium* sp., *Legionella* sp. and *Mycoplasma pneumoniae* were performed by using Bactec 9000 MB automate, BCYE agar plates and SP4 medium as previously described [42]–[44]. Results obtained using routine culture are available in Table S10,S11,S12).

### Quantitative PCR detection

*Mycobacterium* sp., *M. tuberculosis, M. avium* group, *Bosea* sp, *Parachlamydia* sp., *Coxiella burnetii, Chlamedia pneumoniae, Chlamedia psittaci*, *Mycoplasma pneumoniae, Aspergillus* sp., Mimivirus, CMV, HSV, parainfluenza viruses 1 and 3, respiratory syncytial virus, rhinovirus, metapneumovirus, varicella-zoster virus, influenza viruses A and B, enterovirus, and coronaviruses OC-43, 229-E and NL-63 were detected using quantitative PCR. Quantitative PCR was performed using a LightCycler® instrument (Roche Diagnostics, Meylan, France) in conjunction with the QuantiTect Probe PCR Kit. Primers and probes used to identify these microorganisms are reported in table 5. The reaction was performed as previously described [13]. For RNA viruses, RNA was first reverse transcribed using MultiScribe™ Reverse Transcriptase (Applied Biosystems, Courtaboeuf, France) as previously described [13].

### Serology and urinary antigen assay

Sera from patients were tested by Immunofluorescent assay (IFA) for *Coxiella burnetii*, *Bartonella quintana*, *Bartonella henselae, Legionella pneumophila, Legionella anisa* [40], [45], [46]. Viral serologies for adenovirus, cytomegalovirus, herpes simplex, parainfluenza viruses 1 and 3, varicella-zoster virus and, influenza viruses A and B were performed using standard serologic methods (Immunofluorescent assay or enzyme linked immunosorbent assay) [4]. Hemagglutination inhibition, Immunoperoxidase staining and ELISA techniques were used in-house to identify Aspergillosis. *L. pneumophila* antigenuria and CMV pp65 antigenemia were tested for as previously reported [4], [47]. Results obtained using routine serology and antigenemia for viruses and fastidious pathogens are available in Table S13.

### Phylogenetic and statistical analysis

Bacterial and fungal nucleic acid sequences obtained from broad-range primer PCR were aligned with BioEdit program (http://www.mbio.ncsu.edu/BioEdit/bioedit.html) and phylogenetic trees were create with MEGA software version 4.1 using the neighbor-joining method and the Kimura-2 parameter [48]. Species having sequence similarities <98% with those available in GenBank databases were also blasted and classified *in silico* using “Classifier” program in the Ribosomal Database Project (http://rdp.cme.msu.edu/) [49]. Statistical analyses were performed using Chi square test, Fisher's exact test, students t-test or Mantel-Haenszel's Chi square test when appropriate. P values that were less than or equal to 0.05 were considered significant.

### Key words used for literature search

The PubMed database (www.ncbi.nlm.nih.gov/pubmed/) and Google website (http://www.google.fr/) were used to search whether species identified in our study had been previously reported in cases of pneumonia for articles published between 1977 and March 2010, with the combined search term “species name” and “pneumonia”, “lung” or “infection.” Additional articles were identified by hand-searching the references of selected papers. Additional search terms included “microbiology”, “diagnosis”, “16S” and “molecular detection” were used. Only publications in English were considered. Papers in languages other than English were considered only when their abstracts in English were available.

## Supporting Information

Figure S1 **Schematic representation of microorganisms commonly identified in pneumonia and control cohorts, and those only detected in one cohort.** Fungi are shown in rectangles, viruses in octagons, and bacteria in circles. The name of each microorganism is indicated.
(TIF)

Figure S2 **Schematic representation of microorganisms that were commonly identified between each pneumonia form and controls, and those which were detected in only one cohort.** Fungi are shown in rectangles, viruses in octagons, and bacteria in circles. *Actinobacteria* are shown in red, *Bacteroidetes* in yellow, *Chlamydiae* in orange, *Firmicutes* in green, *Fusobacteria* in purple, *Proteobacteria* in blue and *Tenericutes* in sky blue. CAP, community-acquired pneumonia; VAP, ventilator-associated pneumonia; NV ICU-P, non-ventilator ICU pneumonia; AP, aspiration pneumonia; and CS, control subjects.
(TIF)

Figure S3 **Comparison of the bacterial communities found in our study with those found in lung specimens in three previous studies.** Novel phylotypes are not shown. *Actinobacteria* are shown in red, *Bacteroidetes* in yellow, *Chlamydiae* in orange, *Firmicutes* in green, *Fusobacteria* in purple, *Proteobacteria* in blue and *Tenericutes* in sky blue. The name of the first author of each study and the name of each bacterium are indicated. The comparative analysis was conducted using Cytoscape software. VAP, ventilator-associated pneumonia; CF, Cystic fibrosis.
(TIF)

Figure S4 **Molecular methods compared to standard routine culture for bacteria identification.** Bacteria that were identified by molecular methods (A) and by culture (B) performed on BAL samples from all patients are presented according to their classes. Bacterial classes identified by each method are expressed as a percentage. Bacterial classes and their corresponding colors are indicated in the bottom.
(TIF)

Figure S5 **Molecular methods compared to standard cultures for fungi identification.** Fungi that were identified by molecular methods (A) or by culture (B) performed on BAL samples from all patients are presented according to their classes. Fungal classes identified by each method are expressed as a percentage. Fungal classes and their corresponding colours are indicated in the bottom.
(TIF)

Text S1 **BAL culture, blood culture and serology results.**
(DOC)

Data S1 **Detailed data about the relative abundance and richness of each species in their corresponding library.**
(XLSX)

Data S2 **Schematic data about the relative abundance and richness of each species in the corresponding library.**
(XLSX)

Table S1 **Species only detected in BAL from pneumonia patients by molecular assays.**
(DOCX)

Table S2 **Species detected in BAL from pneumonia patients and control subjects by molecular assays.**
(DOCX)

Table S3 **Species only detected in BAL from control subjects by molecular assays.**
(DOCX)

Table S4 **Molecular repertoire of bacteria identified in the present study and their frequency in each cohort.**
(DOCX)

Table S5 **Molecular repertoire of fungi identified in the present study and their frequency in each cohort.**
(DOCX)

Table S6 **Viruses identified by qPCR and their frequency in each cohort.**
(DOCX)

Table S7 **Clinical data in monobacterial, polybacterial, fungal and/or viral and sterile episodes.**
(DOCX)

Table S8 **Comparison between culture and molecular assays performed on BAL specimens.**
(DOCX)

Table S9 **Clinical and sociodemographic data of patients and controls.**
(DOCX)

Table S10 **Repertoire of bacteria identified by culture and their frequency in each cohort.**
(DOCX)

Table S11 **Repertoire of fungi identified by culture and their frequency in each cohort.**
(DOCX)

Table S12 **Repertoire of bacteria identified by blood culture and their frequency in each cohort.**
(DOCX)

Table S13 **Microorganisms identified by serologic testing and their frequency in each cohort.**
(DOCX)
