# Supplementary figures and images for: Repertoire of Intensive Care Unit Pneumonia Microbiota
Source: PLoS One. 2012 Feb 28;7(2):e32486. doi: 10.1371/journal.pone.0032486 (PMC3289664; doi:10.1371/journal.pone.0032486)

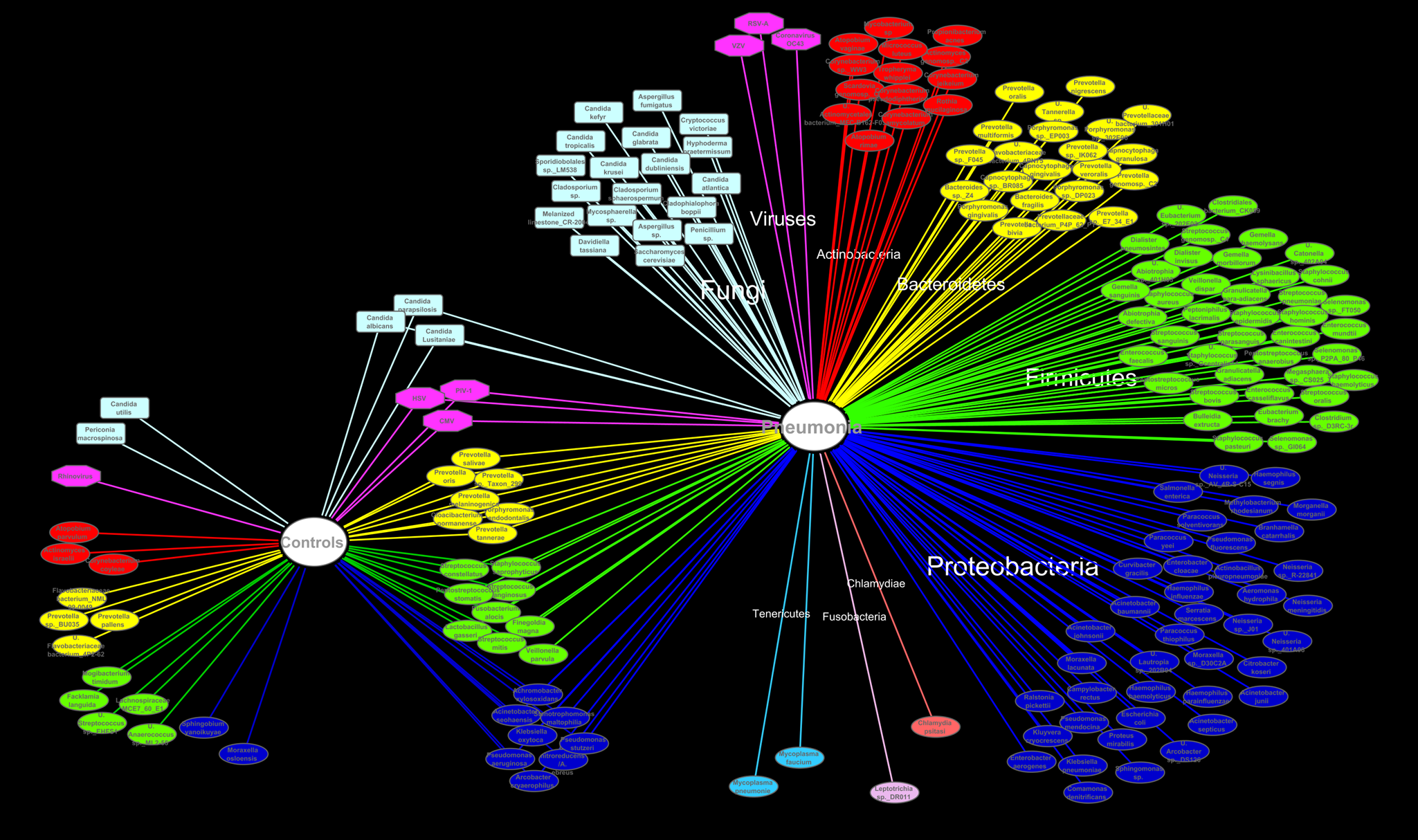

Supplement: Figure S1 — Schematic representation of microorganisms commonly identified in pneumonia and control cohorts, and those only detected in one cohort. Fungi are shown in rectangles, viruses in octagons, and bacteria in circles. The name of each microorganism is indicated. (TIF) [file pone.0032486.s001.tif]

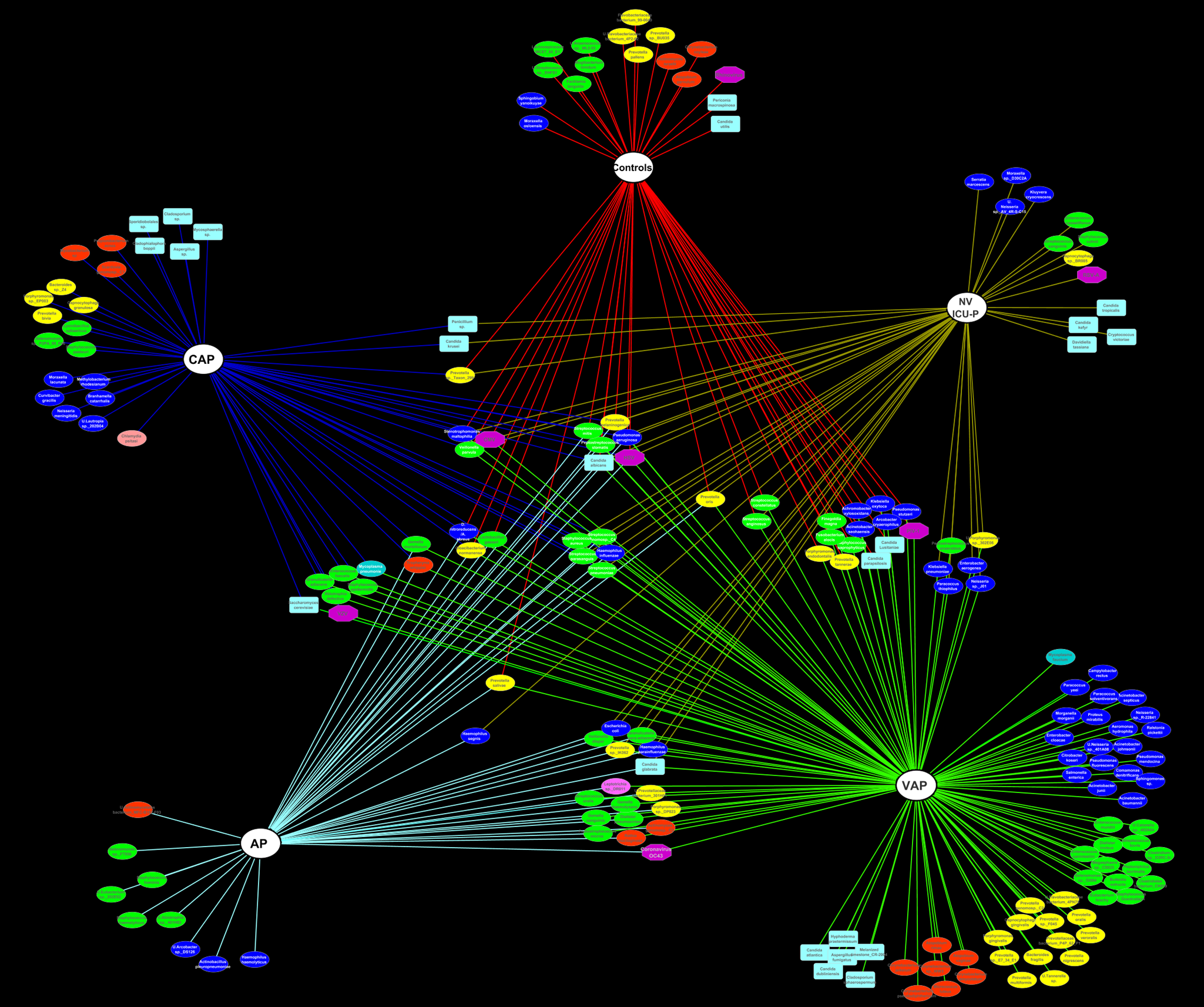

Supplement: Figure S2 — Schematic representation of microorganisms that were commonly identified between each pneumonia form and controls, and those which were detected in only one cohort. Fungi are shown in rectangles, viruses in octagons, and bacteria in circles. Actinobacteria are shown in red, Bacteroidetes in yellow, Chlamydiae in orange, Firmicutes in green, Fusobacteria in purple, Proteobacteria in blue and Tenericutes in sky blue. CAP, community-acquired pneumonia; VAP, ventilator-associated pneumonia; NV ICU-P, non-ventilator ICU pneumonia; AP, aspiration pneumonia; and CS, control subjects. (TIF) [file pone.0032486.s002.tif]

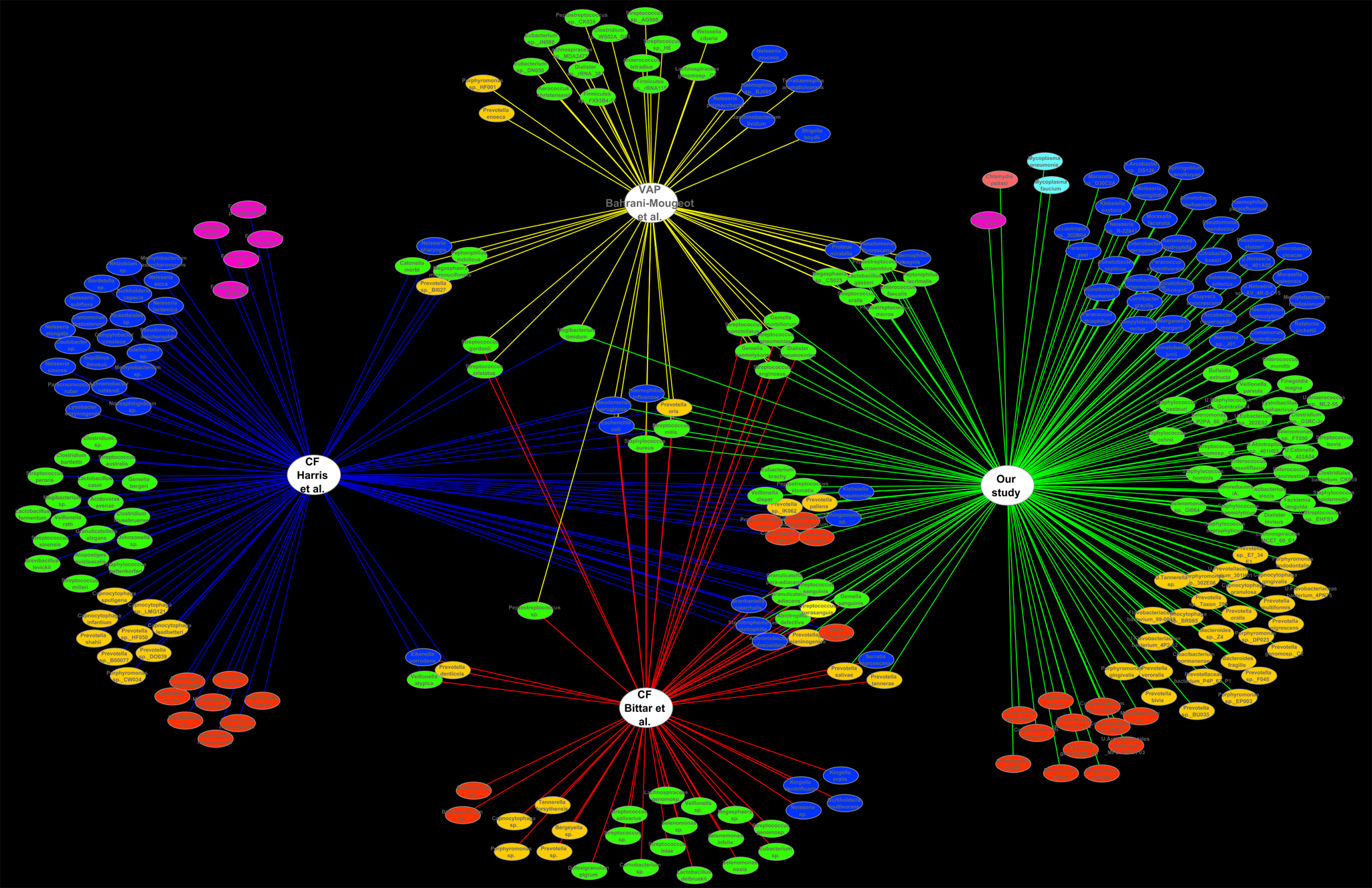

Supplement: Figure S3 — Comparison of the bacterial communities found in our study with those found in lung specimens in three previous studies. Novel phylotypes are not shown. Actinobacteria are shown in red, Bacteroidetes in yellow, Chlamydiae in orange, Firmicutes in green, Fusobacteria in purple, Proteobacteria in blue and Tenericutes in sky blue. The name of the first author of each study and the name of each bacterium are indicated. The comparative analysis was conducted using Cytoscape software. VAP, ventilator-associated pneumonia; CF, Cystic fibrosis. (TIF) [file pone.0032486.s003.tif]

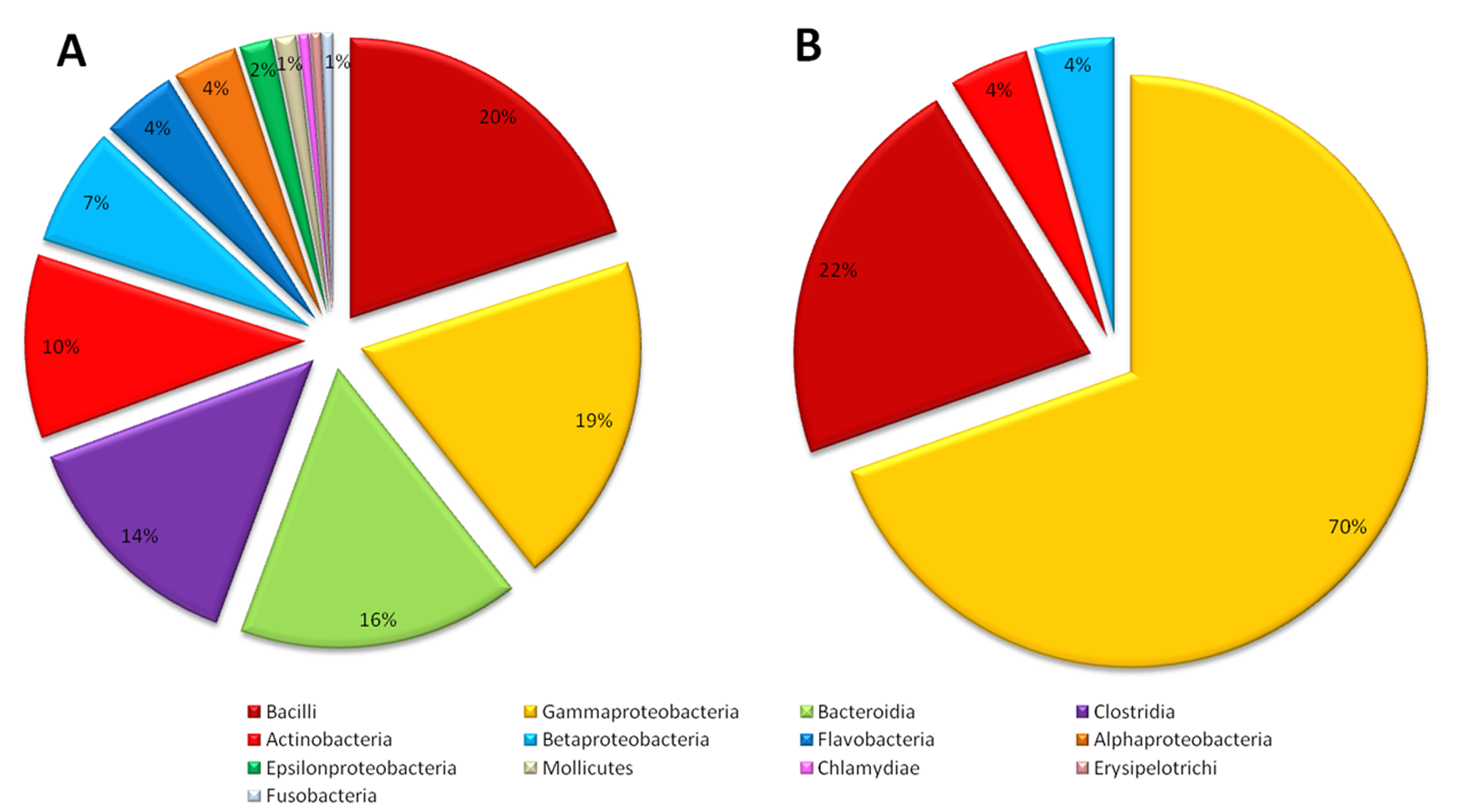

Supplement: Figure S4 — Molecular methods compared to standard routine culture for bacteria identification. Bacteria that were identified by molecular methods (A) and by culture (B) performed on BAL samples from all patients are presented according to their classes. Bacterial classes identified by each method are expressed as a percentage. Bacterial classes and their corresponding colors are indicated in the bottom. (TIF) [file pone.0032486.s004.tif]

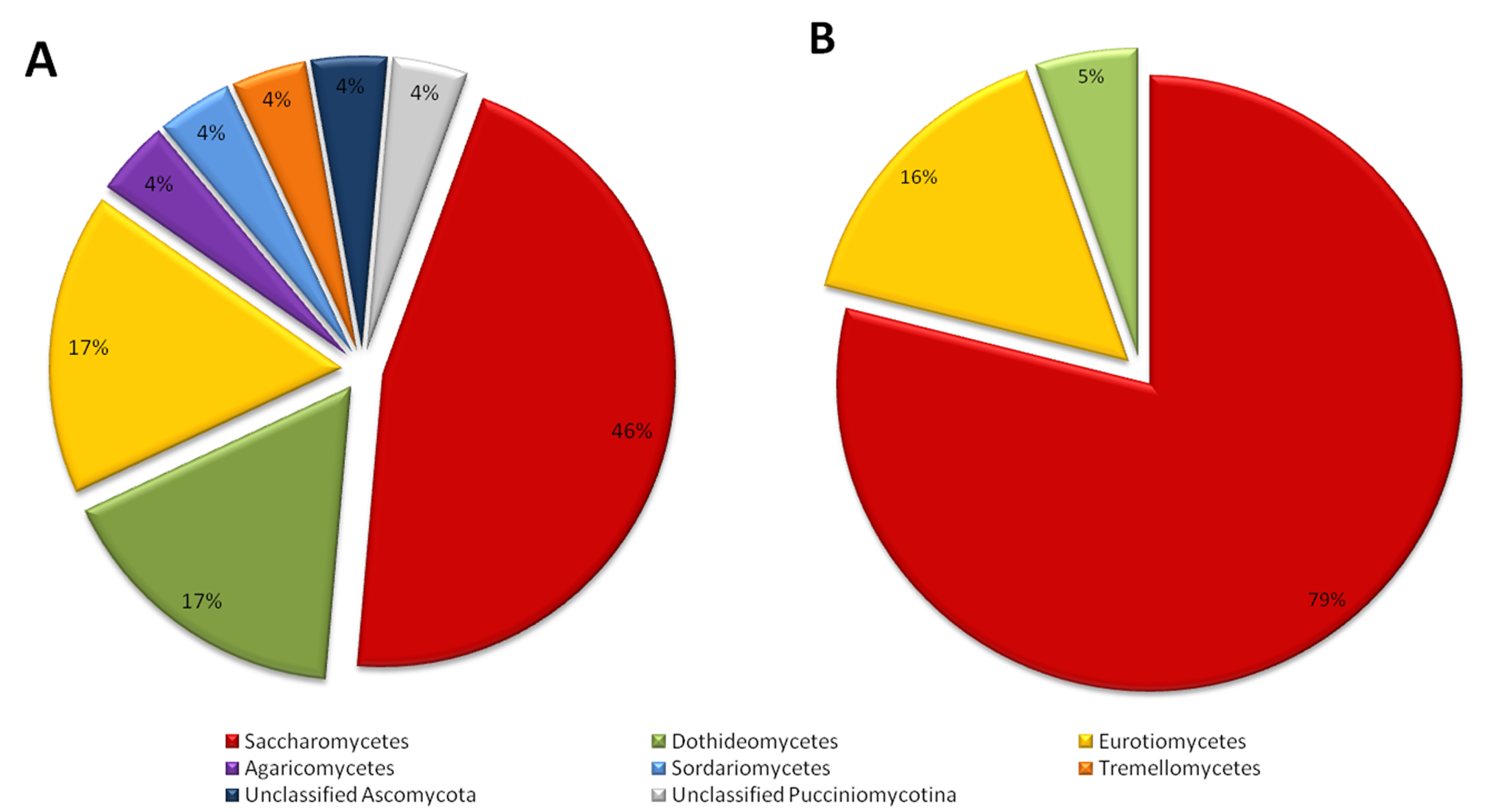

Supplement: Figure S5 — Molecular methods compared to standard cultures for fungi identification. Fungi that were identified by molecular methods (A) or by culture (B) performed on BAL samples from all patients are presented according to their classes. Fungal classes identified by each method are expressed as a percentage. Fungal classes and their corresponding colours are indicated in the bottom. (TIF) [file pone.0032486.s005.tif]
