# Supplementary material for: Repertoire of Intensive Care Unit Pneumonia Microbiota
Source: PLoS One. 2012 Feb 28;7(2):e32486. doi: 10.1371/journal.pone.0032486 (PMC3289664; doi:10.1371/journal.pone.0032486)
Supplement: Text S1 — BAL culture, blood culture and serology results. (DOC) [file pone.0032486.s006.doc]

**Supplementary text**

**BAL culture results**

Results obtained from culture showed that a total of 138 BAL (74%) from pneumonia patients and 17 from controls were positive for bacteria, fungi and/or viruses, of which bacterial identification was not successfully obtained for 24 BAL specimens (21 BAL from pneumonia patients and 3 from controls) due to the presence of complex flora. Bacterial culture was positive for 84 (40%) BAL fluids for one or two bacterium, whereas fungal culture was positive for 89 (42%) BAL fluids for at least one fungus. Culture results showed that pneumonia patients may exhibit up to 7 microorganisms in their BAL fluids. We isolated 23 different bacterial species belonging to *Gammaproteobacteria*, *Bacilli*, *Actinobacteria* and *Betaproteobacteria*, 19 fungi belonging to *Saccharomycetes*, *Eurotiomycetes* and *Dothideomycete* and one virus (Fig. S4 and S5). Bacteria frequently cultured were typical pneumonia pathogens which were (showen in decreasing frequency) *Staphylococcus aureus*, *Pseudomonas aeruginosa*, *Klebsiella pneumoniae*, *Escherichia coli*, *Haemophilus influenzae*, *Streptococcus pneumoniae*, *Stenotrophomonas maltophilia* (Table S10). Bacteria rarely associated to cases of pneumonia were also identified. Four bacteria typical for nosocomial pneumonia (i.e., *Staphylococcus aureus*, *Pseudomonas aeruginosa, Klebsiella oxytoca* and *Achromobacter xilosoxidans*) were also isolated from 4 controls. *Staphylococcus aureus* was isolated from two controls, of whom one was immunocompromised, and the other was immunocompetent. *P. aeruginosa*, *K. oxytoca* and *A. xilosoxidans* were isolated from one immunocompromised control, one immunocompetent control and one immunocompetent control respectively. For fungi, *Candida* species were the most frequently fungal species isolated (Table S11). Of these, *Candida zemplinina*, *Candida inconspicua*, *Candida rugosa* and *Pichia kluyveri* were first isolated from pneumonia specimens in the present study. Herpes simplex virus was isolated from BAL fluid from 2 patients with CAP and one patient with VAP. This virus was also successfully cultured from an immunocompetent control who developed a VAP episode 6 days later. No isolate of *Legionella*, *Mycoplasma* and *Mycobacterium* species was obtained.

**Blood culture results**

Blood cultures were positive for 26 (12%) specimens, of whom 24 specimens were from 24 episodes of pneumonia and 2 were from controls. Bacteria isolated from blood specimens of 10 patients were aligned with the bacteria isolated from BAL culture. A single bacterium was isolated from 22 blood specimens (20 pneumonia blood samples and 2 control blood samples), whereas 4 pneumonia blood specimens were positive for 2 bacteria. Bacteria that usually colonize water, skin, and gastro-intestinal tracts were the most frequently isolated from blood specimens. Results obtained from blood culture showed the identification of 14 different bacterial species, of which 13 species were isolated from pneumonia patients (Table S12). Gram-positive staphylococci and *Pseudomonas aeruginosa* were the most frequent bacteria isolated. *Staphylococcus hominis* was isolated from blood of a patient with ARDS complicating a VAP episode and from a control with ARDS and a history for aspiration pneumonia. *Proteus mirabilis* was isolated only from a control admitted to ICU for more than a month before sampling. All of bacteria identified by blood culture were previously identified in the etiology of pneumonia or bloodstream infections. Fungal and special *Mycobacterium* blood cultures were negative for all patients.

**Serology results**

Results obtained from serology showed that more than 90% our patients exhibited IgG antibody response against HSV, CMV and VZV viruses which is may be due to ancient vaccinations. Thus, to ovoid misleading interpretations, only IgM antibodies were considered in the present study. Serologic tests were definitive, for at least one microorganism, in 52 episodes of pneumonia and 7 controls. Viral serology showed that definitive IgM antibody titers were more frequently detected against CMV an HSV. A significant titer of anti-CMV IgM was detected, as well as a low titer of CMV antigenemia, in one control for whom qPCR targeting CMV on its BAL fluid was negative. Definite HSV IgM titers were detected in 5 pneumonia episodes, of which only two exhibited the virus in their BAL fluids in qPCR. Similar, anti-VZV IgM titers were observed in 3 pneumonia episodes, of which only one was positive for the virus as shown by qPCR in BAL fluids. Seasonal influenza virus A was only observed in pneumonia patients for which attempts to identify the virus in BAL fluids using qPCR was negative. Antibodies response against *Aspergillus* was more frequently diagnosed using ELISA (Table S13). Finally, significant IgM antibodies to *Coxiella burnetii* were detected in one patient who exhibited two nosocomial pneumonia episodes (one NV-ICU-P and one VAP). Significant IgG antibodies against the phase I of the *C. burnetii* (Titer of 1:800, significantly associated with chronic Q fever cases) were detected in a patient with aspiration pneumonia for whom, *H. influenzae* and *S. pneumoniae* bacteria were identified in BAL fluid only by molecular assay.
