# Supplementary material for: Repertoire of Intensive Care Unit Pneumonia Microbiota
Source: PLoS One. 2012 Feb 28;7(2):e32486. doi: 10.1371/journal.pone.0032486 (PMC3289664; doi:10.1371/journal.pone.0032486)
Supplement: Table S1 — Species only detected in BAL from pneumonia patients by molecular assays. (DOCX) [file pone.0032486.s009.docx]

**Table S1: species only detected in BAL from pneumonia patients by molecular assays**

|  | Previousl y reported in pneumonia | Frequency | Not previously reported in pneumonia | Frequency | Unknown phylotypes (N° phylotypes) | Frequency |
| --- | --- | --- | --- | --- | --- | --- |
| Bacteria |  |  |  |  |  |  |
|  | *Staphylococcus aureus* | 17 | *Streptococcus* genomosp. C4 | 9 | *Capnocytophaga* sp. (2) | 2 |
|  | *Streptococcus pneumoniae* | 15 | *Streptococcus parasanguis* | 9 | *Lactobacillus* sp. (2) | 2 |
|  | *Haemophilus influenzae* | 12 | *Granulicatella para-adiacens* | 4 | Unclassified *Bacilli* (2) | 2 |
|  | *Staphylococcus epidermidis* | 8 | *Actinomyces* genomosp. C2 | 3 | Unclassified *Gammaproteobacteria* (2) | 2 |
|  | *Escherichia coli* | 8 | *Dialister invisus* | 3 | Unclassified *Prevotellaceae* (2) | 2 |
|  | *Gemella haemolysans* | 6 | *Gemella sanguinis* | 3 | *Acinetobacter* sp. (1) | 1 |
|  | *Tropheryma whipplei* | 6 | *Neisseria* sp. J01 | 3 | *Anaerovorax* sp. (1) | 1 |
|  | *Haemophilus parainfluenzae* | 5 | *Prevotella* sp. oral clone IK062 | 3 | *Azomonas* sp. (1) | 1 |
|  | *Peptostreptococcus micros* | 5 | *Veillonella dispar* | 3 | *Desulfovibrio* sp. (1) | 1 |
|  | *Klebsiella pneumoniae* | 4 | *Abiotrophia defectiva* | 2 | *Gp3* sp. (1) | 1 |
|  | *Dialister pneumosintes* | 4 | *Comamonas denitrificans* | 2 | *Paraprevotella* sp. (1) | 1 |
|  | *Streptococcus oralis* | 4 | *Granulicatella adiacens* | 2 | *Treponema* sp. (1) | 1 |
|  | *Bacteroides fragilis* | 3 | *Paracoccus solventivorans* | 2 | Unclassified *Alcaligenaceae* (1) | 1 |
|  | *Branhamella catarrhalis* | 3 | *Paracoccus thiophilus* | 2 | Unclassified *Bacteroidales* (1) | 1 |
|  | *Enterobacter aerogenes* | 3 | *Porphyromonas* sp. oral clone DP023 | 2 | Unclassified *Burkholderiales* (1) | 1 |
|  | *Enterococcus faecalis* | 3 | Uncultured *Porphyromonas* sp. clone 302E06 | 2 | Unclassified *Lactobacillales* (1) | 1 |
|  | *Gemella morbillorum* | 3 | Uncultured *Prevotellaceae bacterium* 301H01 | 2 | Unclassified *Microbacteriaceae* (1) | 1 |
|  | *Prevotella oralis* | 3 | *Acinetobacter johnsonii* | 1 | Unclassified *Porphyromonadaceae* (1) | 1 |
|  | *Streptococcus bovis* | 3 | *Acinetobacter septicus* | 1 | Unclassified *Rhizobiales* (1) | 1 |
|  | *Mycoplasma pneumonie* | 2 | *Atopobium vaginae* | 1 | Unclassified *Ruminococcaceae* (1) | 1 |
|  | *Leptotrichia* sp. oral clone DR011 | 2 | *Bacteroides* sp. strain Z4 | 1 | Unclassified *Veillonellaceae* (1) | 1 |
|  | *Mycobacterium* sp | 2 | *Bulleidia extructa* | 1 |  |  |
|  | *Proteus mirabilis* | 2 | *Campylobacter rectus* | 1 |  |  |
|  | *Rothia mucilaginosa* | 2 | *Capnocytophaga granulosa* | 1 |  |  |
|  | *Aeromonas hydrophila* | 2 | *Capnocytophaga* sp. oral clone BR085 | 1 |  |  |
|  | *Citrobacter koseri* | 2 | *Clostridiales bacterium* oral taxon 093 clone CK059 | 1 |  |  |
|  | *Peptostreptococcus anaerobius* | 2 | *Clostridium* sp. D3RC-3r | 1 |  |  |
|  | *Acinetobacter junii* | 2 | *Corynebacterium* sp. WW3 | 1 |  |  |
|  | *Haemophilus segnis* | 2 | *Curvibacter gracilis* | 1 |  |  |
|  | *Prevotella nigrescens* | 2 | *Enterococcus canintestini* | 1 |  |  |
|  | *Staphylococcus cohnii* | 1 | *Enterococcus casseliflavus* | 1 |  |  |
|  | *Staphylococcus haemolyticus* | 1 | *Enterococcus mundtii* | 1 |  |  |
|  | *Acinetobacter baumannii* | 1 | *Lysinibacillus sphaericus* | 1 |  |  |
|  | *Actinobacillus pleuropneumoniae* | 1 | *Methylobacterium rhodesianum* | 1 |  |  |
|  | *Enterobacter cloacae* | 1 | *Moraxella lacunata* | 1 |  |  |
|  | *Micrococcus luteus* | 1 | *Moraxella* sp. D30C2A | 1 |  |  |
|  | *Neisseria meningitidis* | 1 | *Mycoplasma faucium* | 1 |  |  |
|  | *Propionibacterium acnes* | 1 | *Neisseria* sp. R-22841 | 1 |  |  |
|  | *Pseudomonas fluorescens* | 1 | *Porphyromonas* sp. oral clone EP003 | 1 |  |  |
|  | *Ralstonia pickettii* | 1 | *Prevotella bivia* | 1 |  |  |
|  | *Salmonella enterica* | 1 | *Prevotella* genomosp.C2 | 1 |  |  |
|  | *Serratia marcescens* | 1 | *Prevotella multiformis* | 1 |  |  |
|  | *Sphingomonas* sp. | 1 | *Prevotella* sp. E7_34 E1 | 1 |  |  |
|  | *Streptococcus sanguinis* | 1 | *Prevotella* sp. oral clone F045 | 1 |  |  |
|  | *Corynebacterium pseudodiphthericum* | 1 | *Prevotella veroralis* | 1 |  |  |
|  | *Haemophilus haemolyticus* | 1 | *Prevotellaceae bacterium* P4P_62 P1 | 1 |  |  |
|  | *Morganella morganii* | 1 | *Scardovia* genomosp. C1 | 1 |  |  |
|  | *Porphyromonas gingivalis* | 1 | *Selenomonas* sp. oral clone FT050 | 1 |  |  |
|  | *Staphylococcus hominis* | 1 | *Selenomonas* sp. oral clone GI064 | 1 |  |  |
|  | *Atopobium rimae* | 1 | *Selenomonas* sp. oral clone P2PA_80 P46 | 1 |  |  |
|  | *Capnocytophaga gingivalis* | 1 | *Staphylococcus pasteuri* | 1 |  |  |
|  | *Chlamydia psitasi* | 1 | Uncultured *Abiotrophia* sp. clone 401H03 | 1 |  |  |
|  | *Corynebacterium amycolatum* | 1 | Uncultured *Actinomycetales bacterium* clone MFC-B162-F03 | 1 |  |  |
|  | *Corynebacterium jeikeium* | 1 | Uncultured *Arcobacter* sp. clone DS126 | 1 |  |  |
|  | *Eubacterium brachy* | 1 | Uncultured *Flavobacteriaceae bacterium* clone 4PN75 | 1 |  |  |
|  | *Kluyvera cryocrescens* | 1 | Uncultured *Catonella* sp. clone 402A04 |  |  |  |
|  | *Megasphaera* sp. oral clone CS025 | 1 | Uncultured *Eubacterium* sp. clone 202E02 | 1 |  |  |
|  | *Paracoccus yeei* | 1 | Uncultured *Lautropia* sp. 202B04 | 1 |  |  |
|  | *Peptoniphilus lacrimalis* | 1 | Uncultured *Neisseria* sp. clone 401A08 | 1 |  |  |
|  | *Pseudomonas mendocina* | 1 | Uncultured *Neisseria* sp. clone AV_4R-S-C15 | 1 |  |  |
|  |  |  | Uncultured *Staphylococcus* sp. clone Gcentralis14 | 1 |  |  |
|  |  |  | Uncultured *Tannerella* sp. | 1 |  |  |
| Fungi |  |  |  |  |  |  |
|  | *Candida glabrata* | 3 | *Candida atlantica* | 1 |  |  |
|  | *Candida krusei* | 2 | *Cryptococcus victoriae* | 1 |  |  |
|  | *Candida tropicalis* | 2 | *Melanized limestone ascomycete* CR-2004 | 1 |  |  |
|  | *Candida dubliniensis* | 2 | *Mycosphaerella* sp. | 1 |  |  |
|  | *Penicillium* sp. | 2 | *Hyphoderma praetermissum* | 1 |  |  |
|  | *Saccharomyces cerevisiae* | 2 | *Sporidiobolales* sp. LM538 | 1 |  |  |
|  | *Aspergillus.* sp. | 1 |  |  |  |  |
|  | *Aspergillus fumigatus* | 1 |  |  |  |  |
|  | *Cladosporium* sp. | 1 |  |  |  |  |
|  | *Cladosporium sphaerospermum* | 1 |  |  |  |  |
|  | *Candida kefyr /Kluyveromyces marxianus* | 1 |  |  |  |  |
|  | *Cladophialophora boppii* | 1 |  |  |  |  |
|  | *Davidiella tassiana* | 1 |  |  |  |  |
| Viruses |  |  |  |  |  |  |
|  | VZV | 3 |  |  |  |  |
|  | Coronavirus OC43 | 2 |  |  |  |  |
|  | RSV-A | 1 |  |  |  |  |
