# Supplementary material for: Repertoire of Intensive Care Unit Pneumonia Microbiota
Source: PLoS One. 2012 Feb 28;7(2):e32486. doi: 10.1371/journal.pone.0032486 (PMC3289664; doi:10.1371/journal.pone.0032486)
Supplement: Table S2 — Species detected in BAL from pneumonia patients and control subjects by molecular assays. (DOCX) [file pone.0032486.s010.docx]

**Table S2: species detected in BAL from pneumonia patients and control subjects by molecular assays**

|  | Previousl y reported in pneumonia | Frequency | | | Not previously reported in pneumonia | Frequency | | | Unknown phylotypes (N° phylotypes) | Frequency | | |
| --- | --- | --- | --- | --- | --- | --- | --- | --- | --- | --- | --- | --- |
|  |  | Pn | CS | P |  | Pn | CS | P |  | Pn | CS | P |
| Bateria |  |  |  |  |  |  |  |  |  |  |  |  |
|  | *Pseudomonas aeruginosa* | 25 | 2 | 0.43 | *Cloacibacterium normanense* | 13 | 2 | 0.85 | *Anaerococcus* sp. (2) | 1 | 1 | 0.09 |
|  | *Streptococcus mitis* | 20 | 1 | 0.28 | *Diaphorobacter nitroreducens./ Acidovorax ebreus* | 11 | 3 | 0.25 | *Prevotella* sp. (6) | 4 | 2 | 0.10 |
|  | *Prevotella oris* | 14 | 2 | 0.93 | *Peptostreptococcus stomatis* | 7 | 2 | 0.32 |  |  |  |  |
|  | *Prevotella melaninogenica* | 8 | 4 | **0.01** | *Lactobacillus gasseri* | 3 | 1 | 0.41 |  |  |  |  |
|  | *Streptococcus anginosus* | 9 | 3 | 0.14 | *Prevotella salivae* | 2 | 1 | 0.24 |  |  |  |  |
|  | *Pseudomonas stutzeri* | 8 | 2 | 0.41 | *Prevotella* sp. 'Oral Taxon 299' | 2 | 1 | 0.24 |  |  |  |  |
|  | *Stenotrophomonas maltophilia* | 6 | 1 | 0.84 | *Acinetobacter seohaensis* | 1 | 1 | 0.09 |  |  |  |  |
|  | *Streptococcus constellatus* | 6 | 1 | 0.84 | *Finegoldia magna* | 1 | 1 | 0.09 |  |  |  |  |
|  | *Achromobacter xylosoxidans* | 4 | 1 | 0.57 | *Fusobacterium alocis* | 1 | 1 | 0.09 |  |  |  |  |
|  | *Veillonella parvula* | 4 | 1 | 0.57 | *Porphyromonas endodontalis* | 1 | 1 | 0.09 |  |  |  |  |
|  | *Arcobacter cryaerophilus* | 2 | 2 | **0.01** | *Prevotella tannerae* | 1 | 1 | 0.09 |  |  |  |  |
|  | *Klebsiella oxytoca* | 2 | 1 | 0.24 |  |  |  |  |  |  |  |  |
|  | *Staphylococcus saprophyticus* | 1 | 1 | 0.09 |  |  |  |  |  |  |  |  |
| Fungi |  |  |  |  |  |  |  |  |  |  |  |  |
|  | *Candida albicans* | 14 | 2 | 0.93 |  |  |  |  |  |  |  |  |
|  | *Candida parapsilosis* | 3 | 1 | 0.41 |  |  |  |  |  |  |  |  |
|  | *Candida. Lusitaniae* | 2 | 1 | 0.24 |  |  |  |  |  |  |  |  |
| Viruses |  |  |  |  |  |  |  |  |  |  |  |  |
|  | HSV | 51 | 7 | 0.96 |  |  |  |  |  |  |  |  |
|  | CMV | 31 | 3 | 0.54 |  |  |  |  |  |  |  |  |
|  | PIV-1 | 3 | 1 | 0.41 |  |  |  |  |  |  |  |  |

Pn, pneumonia; CS, control subjects.
