# Supplementary material for: Repertoire of Intensive Care Unit Pneumonia Microbiota
Source: PLoS One. 2012 Feb 28;7(2):e32486. doi: 10.1371/journal.pone.0032486 (PMC3289664; doi:10.1371/journal.pone.0032486)
Supplement: Table S3 — Species only detected in BAL from control subjects by molecular assays. (DOCX) [file pone.0032486.s011.docx]

**Table S3: species only detected in BAL from control subjects by molecular assays**

|  | Previousl y reported in pneumonia | Frequency | Not previously reported in pneumonia | Frequency | Unknown phylotypes (N° phylotypes) | Frequency |
| --- | --- | --- | --- | --- | --- | --- |
| Bacteria |  |  |  |  |  |  |
|  | *Actinomyces israelii* | 1 | *Lachnospiraceae*  MCE7_60 E1 | 3 | *Paenibacillus* sp. (1) | 1 |
|  | *Mogibacterium timidum* | 1 | *Atopobium parvulum* | 2 | Unclassified *Actinomycetales* (1) | 1 |
|  | *Moraxella osloensis* | 1 | *Prevotella pallens* | 2 |  |  |
|  |  |  | *Corynebacterium coyleae* | 1 |  |  |
|  |  |  | *Facklamia languida* | 1 |  |  |
|  |  |  | *Flavobacteriaceae bacterium* NML 99-0049 | 1 |  |  |
|  |  |  | *Prevotella* sp. BU035 | 1 |  |  |
|  |  |  | *Sphingobium yanoikuyae* | 1 |  |  |
|  |  |  | Uncultured *Anaerococcus* sp. ML2-55 | 1 |  |  |
|  |  |  | Uncultured *Flavobacteriaceae bacterium 4P2-62* | 1 |  |  |
|  |  |  | Uncultured *Streptococcus* sp. EHFS1 | 1 |  |  |
| Fungi |  |  |  |  |  |  |
|  |  |  | *Candida utilis* | 2 |  |  |
|  |  |  | *Periconia macrospinosa* | 1 |  |  |
| Viruses |  |  |  |  |  |  |
|  | Rhinovirus | 1 |  |  |  |  |

CAP, community-associated pneumonia; VAP, ventilator-associated pneumonia; NV ICU-P, non-ventilator ICU pneumonia; AP, aspiration pneumonia; CS, control subjects.
