# Supplementary material for: Repertoire of Intensive Care Unit Pneumonia Microbiota
Source: PLoS One. 2012 Feb 28;7(2):e32486. doi: 10.1371/journal.pone.0032486 (PMC3289664; doi:10.1371/journal.pone.0032486)
Supplement: Table S4 — Molecular repertoire of bacteria identified in the present study and their frequency in each cohort. (DOCX) [file pone.0032486.s012.docx]

**Table S4: molecular repertoire of bacteria identified in the present study and their frequency in each cohort**

| *Bacteria* | *Gram* | *Aero/Anaero* | *Origin* | *Frequency* (n=210) | *Frequency in pneumonia cohorts* | | | | *Frequency in pneumonia Vs CS* | | |
| --- | --- | --- | --- | --- | --- | --- | --- | --- | --- | --- | --- |
|  |  |  |  |  | CAP (n=32) | VAP (n=106) | NV ICU-P (n=22) | AP (n=25) | Pneumonia patients (n=185) | CS (n=25) | P value |
| *Pseudomonas aeruginosa* | - | Aero | Oral flora, water, gastrointestinal tract, environment | 27 (13%) | 4 (13%) | 16 (15%) | 5 (23%) | 0 | 25 (14%) | 2 (8%) | 0.43 |
| *Streptococcus mitis* | + | Facultative | Oral flora, dental plaque | 21 (10%) | 2 (6%) | 13 (12%) | 2 (9%) | 3 (12%) | 20 (11%) | 1 (4%) | 0.28 |
| *Staphylococcus aureus* | + | Facultative | Skin, nasopharyngeal mucosa | 17 (8%) | 2 (6%) | 10 (9%) | 3 (14%) | 2 (4%) | 17 (9%) | 0 | 0.23 |
| *Streptococcus pneumoniae* | + | Facultative | Nasopharyngeal mucosa | 15 (7%) | 2 (6%) | 6 (6%) | 1 (5%) | 6 (24%) | 15 (8%) | 0 | 0.23 |
| *Cloacibacterium normanense* | - | Facultative | Wastewater | 15 (7%) | 2 (6%) | 9 (8%) | 0 | 2 (8%) | 13 (7%) | 2 (8%) | 0.85 |
| *Diaphorobacter nitroreducens./ Acidovorax ebreus* | - | Facultative | Activated sludge/ groundwater | 14 (7%) | 2 (6%) | 8 (8%) | 0 | 1 (4%) | 11 (6%) | 3 (12%) | 0.25 |
| *Prevotella oris* | - | Anaero | Oral flora, dental plaque | 14 (6%) | 0 | 10 (8%) | 1 (5%) | 1 (4%) | 12 (6%) | 2 (8%) | 0.93 |
| *Prevotella melaninogenica* | - | Anaero | Oral flora, dental plaque | 12 (6%) | 1 (3%) | 3 (3%) | 1 (5%) | 3 (12%) | 8 (4%) | 4 (16%) | **0.01** |
| *Haemophilus influenzae* | - | Facultative | Oral flora, dental plaque | 12 (6%) | 4 (13%) | 2 (2%) | 3 (14%) | 3 (12%) | 12 (6%) | 0 | 0.18 |
| *Streptococcus anginosus* | + | Facultative | Oral flora, gastrointestinal tract, genital tract | 12 (6%) | 0 | 8 (8%) | 1 (5%) | 0 | 9 (5%) | 3 (12%) | 0.14 |
| *Pseudomonas stutzeri* | - | Aero | Soil, water environments | 10 (5%) | 0 | 8 (8%) | 0 | 0 | 8 (4%) | 2 (8%) | 0.41 |
| *Streptococcus* genomosp. C4 | + | Facultative | Oral flora, dental plaque | 9 (4%) | 1 (3%) | 3 (3%) | 3 (14%) | 2 (8%) | 9 (5%) | 0 | 0.60 |
| *Streptococcus parasanguinis* | + | Facultative | Oral flora, dental plaque | 9 (4%) | 2 (6%) | 3 (3%) | 1 (5%) | 3 (12%) | 9 (5%) | 0 | 0.60 |
| *Peptostreptococcus stomatis* | + | Anaero | Oral flora, dental plaque | 9 (4%) | 1 (3%) | 2 (2%) | 1 (5%) | 3 (12%) | 7 (4%) | 2 (8%) | 0.32 |
| *Escherichia coli* | - | Aero | Water , Feces, gastrointestinal tract | 8 (4%) | 0 | 5 (5%) | 1 (5%) | 2 (8%) | 8 (4%) | 0 | 0.60 |
| *Staphylococcus epidermidis* | + | Facultative | Skin, nasopharyngeal mucosa | 8 (4%) | 2 (6%) | 6 (6%) | 0 | 0 | 8 (4%) | 0 | 0.60 |
| *Stenotrophomonas maltophilia* | - | Aero | Water, soil | 7 (3%) | 1 (3%) | 4 (4%) | 1 (5%) | 0 | 6 (3%) | 1 (4%) | 0.84 |
| *Gemella haemolysans* | + | Aero | oral flora, dental plaque, nasopharyngeal mucosa | 6 (3%) | 0 | 3 (3%) | 0 | 3 (12%) | 6 (3%) | 0 | 1 |
| *Streptococcus constellatus* | + | Facultative | Oral flora | 7 (3%) | 0 | 5 (4%) | 1 (5%) | 0 | 6 (3%) | 1 (4%) | 0.84 |
| *Tropheryma whipplei* | + | Aero | Environment* | 6 (3%) | 1 (3%) | 3 (3%) | 0 | 2 (8%) | 6 (3%) | 0 | 1 |
| *Achromobacter xylosoxidans* | - | Aero | Water, environment | 5 (2%) | 0 | 4 (4%) | 0 | 0 | 4 (2%) | 1 (4%) | 0.57 |
| *Peptostreptococcus micros* | + | Anaero | Oral flora | 5 (2%) | 0 | 4 (4%) | 0 | 1 (4%) | 5 (3%) | 0 | 1 |
| *Veillonella parvula* | - | Anaero | Oral flora, gastrointestinal tract, vaginal flora | 5 (2%) | 1 (3%) | 2 (2%) | 1 (5%) | 0 | 4 (2%) | 1 (4%) | 0.57 |
| *Haemophilus parainfluenzae* | - | Facultative | Oral flora, dental plaque | 5 (2%) | 0 | 2 (2%) | 1 (5%) | 2 (8%) | 5 (3%) | 0 | 1 |
| *Arcobacter cryaerophilus* | - | Facultative | Water | 4 (2%) | 0 | 2 (2%) | 0 | 0 | 2 (1%) | 2 (8%) | **0.01** |
| *Dialister pneumosintes* | - | Anaero | Oral flora, dental plaque | 4 (2%) | 0 | 3 (3%) | 0 | 1 (4%) | 4 (2%) | 0 | 1 |
| *Granulicatella para-adiacens* | + | Facultative | Oral flora | 4 (2%) | 0 | 2 (2%) | 1 (5%) | 1 (4%) | 4 (2%) | 0 | 1 |
| *Lactobacillus gasseri* | + | Anaero | Gastrointestinal tract | 4 (2%) | 1 (3%) | 1 (1%) | 0 | 1 (4%) | 3 (2%) | 1 (4%) | 0.41 |
| *Streptococcus oralis* | + | Facultative | Oral flora, dental plaque | 4 (2%) | 0 | 1 (1%) | 0 | 3 (12%) | 4 (2%) | 0 | 1 |
| *Klebsiella pneumoniae* | - | Facultative | Water, soil, environment | 4 (2%) | 0 | 3 (3%) | 1 (5%) | 0 | 4 (2%) | 0 | 1 |
| *Enterobacter aerogenes* | - | Facultative | Water, soil, gastrointestinal tract | 3 (1%) | 0 | 2 (2%) | 1 (5%) | 0 | 3 (2%) | 0 | 1 |
| *Gemella sanguinis* | + | Facultative | Oral flora | 3 (1%) | 0 | 1 (1%) | 0 | 2 (8%) | 3 (2%) | 0 | 1 |
| *Actinomyces* genomosp. C2 | + | Facultative | Oral flora | 3 (1%) | 0 | 2 (2%) | 0 | 1 (4%) | 3 (2%) | 0 | 1 |
| *Bacteroides fragilis* | - | Anaero | Gastrointestinal flora | 3 (1%) | 0 | 3 (3%) | 0 | 0 | 3 (2%) | 0 | 1 |
| *Branhamella catarrhalis* | - | Aero | Oral flora, nasopharyngeal mucosa | 3 (1%) | 3 (9%) | 0 | 0 | 0 | 3 (2%) | 0 | 1 |
| *Dialister invisus* | - | Anaero | Oral flora, dental plaque | 3 (1%) | 0 | 3 (3%) | 0 | 0 | 3 (2%) | 0 | 1 |
| *Enterococcus faecalis* | + | Facultative | Soil,, water, intestinal flora | 3 (1%) | 1 (3%) | 2 (2%) | 0 | 0 | 3 (2%) | 0 | 1 |
| *Gemella morbillorum* | + | Facultative | Oral flora | 3 (1%) | 1 (3%) | 1 (1%) | 0 | 1 (4%) | 3 (2%) | 0 | 1 |
| *Klebsiella oxytoca* | - | Facultative | Water, soil, environment | 3 (1%) | 0 | 2 (2%) | 0 | 0 | 2 (1%) | 1 (4%) | 0.24 |
| *Lachnospiraceae bacterium* oral clone MCE7_60 E1 | ND | ND | Oral flora | 3 (1%) | 0 | 0 | 0 | 0 | 0 | 3 (12%) | **0.001** |
| *Neisseria* sp. J01 | - | Aero | Oral flora | 3 (1%) | 0 | 1 (1%) | 2 (9%) | 0 | 3 (2%) | 0 | 1 |
| *Prevotella oralis* | - | Anaero | Oral flora | 3 (1%) | 0 | 3 (3%) | 0 | 0 | 3 (2%) | 0 | 1 |
| *Prevotella* sp. ‘Oral Taxon 299’ | - | Anaero | Oral flora | 3 (1%) | 1 (3%) | 0 | 1 (5%) | 0 | 2 (1%) | 1 (4%) | 0.24 |
| *Prevotella* sp. Oral clone IK062 | - | Anaero | Oral flora | 3 (1%) | 0 | 1 (1%) | 1 (5%) | 1 (4%) | 3 (2%) | 0 | 1 |
| *Prevotella salivae* | - | Anaero | Oral flora | 3 (1%) | 0 | 1 (1%) | 0 | 1 (4%) | 2 (1%) | 1 (4%) | 0.24 |
| *Streptococcus bovis* | + | Facultative | Alimentary tract of ruminants | 3 (1%) | 0 | 3 (3%) | 0 | 0 | 3 (2%) | 0 | 1 |
| *Veillonella dispar* | - | Anaero | Oral flora, gastrointestinal tract, dental plaque, vaginal flora | 3 (1%) | 0 | 1 (1%) | 1 (5%) | 1 (4%) | 3 (2%) | 0 | 1 |
| *Abiotrophia efective* | + | Facultative | Oral flora, gastrointestinal tract, urogenital tract | 2 (1%) | 1 (3%) | 1 (1%) | 0 | 0 | 2 (1%) | 0 | 1 |
| *Acinetobacter junii* | - | Aero | Water, soil, skin | 2 (1%) | 0 | 2 (2%) | 0 | 0 | 2 (1%) | 0 | 1 |
| *Acinetobacter seohaensis* | - | Aero | Water, soil | 2 (1%) | 0 | 1 (1%) | 0 | 0 | 1 (0.5%) | 1 (4%) | 0.09 |
| *Aeromonas hydrophila* | - | Facultative | Water, soil | 2 (1%) | 0 | 2 (2%) | 0 | 0 | 2 (1%) | 0 | 1 |
| *Atopobium parvulum* | + | Anaero | Oral flora | 2 (1%) | 0 | 0 | 0 | 0 | 0 | 2 (8%) | **0.01** |
| *Citrobacter koseri* | - | Facultative | Gastrointestinal tract, feces, water, soil | 2 (1%) | 0 | 2 (2%) | 0 | 0 | 2 (1%) | 0 | 1 |
| *Finegoldia magna* | + | Anaero | Skin, astrointestinal tracts, mouth mucosa | 2 (1%) | 0 | 1 (1%) | 0 | 0 | 1 (0.5%) | 1 (4%) | 0.09 |
| *Fusobacterium alocis* | - | Anaero | Oral flora, dental plaque | 2 (1%) | 0 | 1 (1%) | 0 | 0 | 1 (0.5%) | 1 (4%) | 0.09 |
| *Granulicatella adiacens* | + | Facultative | Oral flora, gastrointestinal tract, urogenital tract | 2 (1%) | 1 (3%) | 1 (1%) | 0 | 0 | 2 (1%) | 0 | 1 |
| *Haemophilus segnis* | - | Facultative | Oral flora | 2 (1%) | 0 | 0 | 1 (5%) | 1 (4%) | 2 (1%) | 0 | 1 |
| *Paracoccus solventivorans* | - | Facultative | Soil | 2 (1%) | 0 | 2 (2%) | 0 | 0 | 2 (1%) | 0 | 1 |
| *Peptostreptococcus anaerobius* | + | Anaero | Oral flora, dental plaque | 2 (1%) | 0 | 1 (1%) | 1 (5%) | 0 | 2 (1%) | 0 | 1 |
| *Prevotella nigrescens* | - | Anaero | Oral flora, dental plaque | 2 (1%) | 0 | 2 (2%) | 0 | 0 | 2 (1%) | 0 | 1 |
| *Porphyromonas endodontalis* | - | Anaero | Oral flora, dental plaque | 2 (1%) | 0 | 1 (1%) | 0 | 0 | 1 (0.5%) | 1 (4%) | 0.09 |
| *Porphyromonas* sp. Oral clone DP023 | - | Anaero | Oral flora | 2 (1%) | 0 | 1 (1%) | 0 | 1 (4%) | 2 (1%) | 0 | 1 |
| *Prevotella pallens* | - | Anaero | Oral flora, dental plaque | 2 (1%) | 0 | 0 | 0 | 0 | 0 | 2 (8%) | **0.01** |
| *Prevotella tannerae* | - | Anaero | Oral flora, dental plaque | 2 (1%) | 0 | 1 (1%) | 0 | 0 | 1 (0.5%) | 1 (4%) | 0.09 |
| *Proteus mirabilis* | - | Facultative | Soil, water, gastrointestinal tract | 2 (1%) | 0 | 2 (2%) | 0 | 0 | 2 (1%) | 0 | 1 |
| *Rothia mucilaginosa* | + | Aero | Oral flora | 2 (1%) | 0 | 1 (1%) | 0 | 1 (4%) | 2 (1%) | 0 | 1 |
| *Staphylococcus saprophyticus subsp. Saprophyticus* | + | Facultative | Urinary tract, environment | 2 (1%) | 0 | 1 (1%) | 0 | 0 | 1 (0.5%) | 1 (4%) | 0.09 |
| Uncultured *Porphyromonas* sp. Clone 302E06 | - | Anaero | Dental plaque | 2 (1%) | 0 | 1 (1%) | 1 (5%) | 0 | 2 (1%) | 0 | 1 |
| Uncultured *Prevotellaceae bacterium* 301H01 | - | Anaero | Dental plaque | 2 (1%) | 0 | 1 (1%) | 0 | 1 (4%) | 2 (1%) | 0 | 1 |
| *Comamonas denitrificans* | - | Aero | Wastewater, activated sludge | 2 (1%) | 0 | 2 (2%) | 0 | 0 | 2 (1%) | 0 | 1 |
| *Leptotrichia* sp. Oral clone DR011 | - | Anaero | Oral flora, dental plaque | 2 (1%) | 0 | 1 (1%) | 0 | 1 (4%) | 2 (1%) | 0 | 1 |
| *Paracoccus thiophilus* | - | Facultative | Wastewater, soil | 2 (1%) | 0 | 1 (1%) | 1 (5%) | 0 | 2 (1%) | 0 | 1 |
| *Mycobacterium* sp | + | Facultative | Water, soil | 2 (1%) | 2 (6%) | 0 | 0 | 0 | 2 (1%) | 0 | 1 |
| *Mycoplasma pneumoniae* | -/+* | Facultative | Environment | 2 (1%) | 1 (3%) | 1 (1%) | 0 | 0 | 2 (1%) | 0 | 1 |
| *Streptococcus sanguinis* | + | Facultative | Oral flora, dental plaque | 1 (<1%) | 0 | 0 | 1 (5%) | 0 | 1 (<1%) | 0 | 1 |
| *Acinetobacter johnsonii* | - | Aero | Water, soil, skin | 1 (<1%) | 0 | 1 (1%) | 0 | 0 | 1 (<1%) | 0 | 1 |
| *Actinobacillus pleuropneumoniae* | - | Aero | Oral cavity | 1 (<1%) | 0 | 0 | 0 | 1 (4%) | 1 (<1%) | 0 | 1 |
| *Acinetobacter baumannii* | - | Aero | Water, soil | 1 (<1%) | 0 | 1 (1%) | 0 | 0 | 1 (<1%) | 0 | 1 |
| *Acinetobacter septicus* | - | Aero | Water, soil | 1 (<1%) | 0 | 1 (1%) | 0 | 0 | 1 (<1%) | 0 | 1 |
| *Actinomyces israelii* | + | Anaero | Oral flora, mucosa | 1 (<1%) | 0 | 0 | 0 | 0 | 0 | 1 (4%) | 0.11 |
| *Atopobium rimae* | + | Anaero | Dental plaque | 1 (<1%) | 0 | 1 (1%) | 0 | 0 | 1 (<1%) | 0 | 1 |
| *Atopobium vaginae* | + | Anaero | Vaginal flora | 1 (<1%) | 0 | 1 (1%) | 0 | 0 | 1 (<1%) | 0 | 1 |
| *Bacteroides* sp. Strain Z4 | - | Anaero | Wastewater | 1 (<1%) | 1 (3%) | 0 | 0 | 0 | 1 (<1%) | 0 | 1 |
| *Bulleidia extructa* | + | Anaero | Oral flora, dental plaque | 1 (<1%) | 0 | 1 (1%) | 0 | 0 | 1 (<1%) | 0 | 1 |
| *Campylobacter rectus* | - | Anaero | Oral flora, dental plaque | 1 (<1%) | 0 | 1 (1%) | 0 | 0 | 1 (<1%) | 0 | 1 |
| *Capnocytophaga gingivalis* | - | Aero | Oral flora, dental plaque | 1 (<1%) | 0 | 1 (1%) | 0 | 0 | 1 (<1%) | 0 | 1 |
| *Capnocytophaga granulosa* | - | Aero | Oral flora, dental plaque | 1 (<1%) | 1 (3%) | 0 | 0 | 0 | 1 (<1%) | 0 | 1 |
| *Capnocytophaga* sp. Oral clone BR085 | - | Aero | Oral flora, dental plaque | 1 (<1%) | 0 | 0 | 1 (5%) | 0 | 1 (<1%) | 0 | 1 |
| *Chlamydia psittasi* | - | Facultative | Fowl feces | 1 (<1%) | 1 (3%) | 0 | 0 | 0 | 1 (<1%) | 0 | 1 |
| *Clostridiales bacterium* oral taxon 093 clone CK059 | + | Anaero | Oral flora | 1 (<1%) | 0 | 1 (1%) | 0 | 0 | 1 (<1%) | 0 | 1 |
| *Clostridium* sp. D3RC-3r | + | Anaero | Oral flora | 1 (<1%) | 0 | 1 (1%) | 0 | 0 | 1 (<1%) | 0 | 1 |
| *Corynebacterium amycolatum* | + | Facultative | Oral flora, Skin, mucosa | 1 (<1%) | 0 | 1 (1%) | 0 | 0 | 1 (<1%) | 0 | 1 |
| *Corynebacterium coyleae* | + | Facultative | Skin, mucosa | 1 (<1%) | 0 | 0 | 0 | 0 | 0 | 1 (4%) | 0.11 |
| *Corynebacterium jeikeium* | + | Aero | Skin, nasopharyngeal mucosa | 1 (<1%) | 0 | 1 (1%) | 0 | 0 | 1 (<1%) | 0 | 1 |
| *Corynebacterium pseudodiphthericum* | + | Facultative | Skin, nasopharyngeal mucosa | 1 (<1%) | 0 | 1 (1%) | 0 | 0 | 1 (<1%) | 0 | 1 |
| *Corynebacterium* sp. WW3 | + | Facultative | Wastewater | 1 (<1%) | 0 | 1 (1%) | 0 | 0 | 1 (<1%) | 0 | 1 |
| *Curvibacter gracilis* | - | Aero | Water | 1 (<1%) | 1 (3%) | 0 | 0 | 0 | 1 (<1%) | 0 | 1 |
| *Enterobacter cloacae* | - | Facultative | Skin, plant, water, soil, gastrointestinal tract | 1 (<1%) | 0 | 1 (1%) | 0 | 0 | 1 (<1%) | 0 | 1 |
| *Enterococcus canintestini* | + | Facultative | Dog feces | 1 (<1%) | 0 | 1 (1%) | 0 | 0 | 1 (<1%) | 0 | 1 |
| *Enterococcus casseliflavus* | + | Facultative | Cheese, biliary tract | 1 (<1%) | 0 | 0 | 1 (5%) | 0 | 1 (<1%) | 0 | 1 |
| *Enterococcus mundtii* | + | Facultative | Plant, soil, water | 1 (<1%) | 0 | 1 (1%) | 0 | 0 | 1 (<1%) | 0 | 1 |
| *Eubacterium brachy* | + | Anaero | Oral flora, dental plaque | 1 (<1%) | 0 | 1 (1%) | 0 | 0 | 1 (<1%) | 0 | 1 |
| *Mogibacterium timidum* | + | Anaero | Oral flora, dental plaque | 1 (<1%) | 0 | 0 | 0 | 0 | 0 | 1 (4%) | 0.11 |
| *Facklamia languida* | + | Facultative | Urogenital tract* | 1 (<1%) | 0 | 0 | 0 | 0 | 0 | 1 (4%) | 0.11 |
| *Flavobacteriaceae* bacterium NML no.99-0049 | - | Aero | Soil, water* | 1 (<1%) | 0 | 0 | 0 | 0 | 0 | 1 (4%) | 0.11 |
| *Haemophilus haemolyticus* | - | Facultative | Oral flora | 1 (<1%) | 0 | 0 | 0 | 1 (4%) | 1 (<1%) | 0 | 1 |
| *Kluyvera cryocrescens* | - | Aero | Oral flora, soil, water, gut | 1 (<1%) | 0 | 0 | 1 (5%) | 0 | 1 (<1%) | 0 | 1 |
| *Megasphaera* sp. Oral clone CS025 | - | Anaero | Dental plaque | 1 (<1%) | 0 | 1 (1%) | 0 | 0 | 1 (<1%) | 0 | 1 |
| *Methylobacterium rhodesianum* | - | Aero | Oral flora, soil, water | 1 (<1%) | 1 (3%) | 0 | 0 | 0 | 1 (<1%) | 0 | 1 |
| *Micrococcus luteus* | + | Facultative | Skin, oral flora, nasopharyngeal mucosa, environment, water | 1 (<1%) | 0 | 1 (1%) | 0 | 0 | 1 (<1%) | 0 | 1 |
| *Moraxella lacunata* | - | Aero | Oral flora, nasopharyngeal mucosa, water | 1 (<1%) | 1 (3%) | 0 | 0 | 0 | 1 (<1%) | 0 | 1 |
| *Moraxella* sp. D30C2A | - | Aero | Water | 1 (<1%) | 0 | 0 | 1 (5%) | 0 | 1 (<1%) | 0 | 1 |
| *Moraxella osloensis* | - | Aero | Skin, nasopharyngeal mucosa | 1 (<1%) | 0 | 0 | 0 | 0 | 0 | 1 (4%) | 0.11 |
| *Morganella morganii* | - | Facultative | Gastrointestinal tract | 1 (<1%) | 0 | 1 (1%) | 0 | 0 | 1 (<1%) | 0 | 1 |
| *Mycoplasma faucium* | - | Facultative | Oral flora, | 1 (<1%) | 0 | 1 (1%) | 0 | 0 | 1 (<1%) | 0 | 1 |
| *Neisseria meningitidis* | - | Aero | Nasopharynx, environment* | 1 (<1%) | 1 (3%) | 0 | 0 | 0 | 1 (<1%) | 0 | 1 |
| *Neisseria* sp. R-22841 | - | Aero | Water , oral mucosa | 1 (<1%) | 0 | 1 (1%) | 0 | 0 | 1 (<1%) | 0 | 1 |
| *Paracoccus yeei* | - | Aero | Soil, brines | 1 (<1%) | 0 | 1 (1%) | 0 | 0 | 1 (<1%) | 0 | 1 |
| *Peptoniphilus lacrimalis* | + | Anaero | Vaginal tract | 1 (<1%) | 0 | 1 (1%) | 0 | 0 | 1 (<1%) | 0 | 1 |
| *Porphyromonas gingivalis* | - | Anaero | Oral flora, dental plaque | 1 (<1%) | 0 | 1 (1%) | 0 | 0 | 1 (<1%) | 0 | 1 |
| *Porphyromonas* sp. Oral clone EP003 | - | Anaero | Dental plaque | 1 (<1%) | 1 (3%) | 0 | 0 | 0 | 1 (<1%) | 0 | 1 |
| *Prevotella bivia* | - | Anaero | Oral flora, dental plaque, genital tract | 1 (<1%) | 1 (3%) | 0 | 0 | 0 | 1 (<1%) | 0 | 1 |
| *Prevotella* genomosp. C2 | - | Anaero | Dental plaque | 1 (<1%) | 0 | 1 (1%) | 0 | 0 | 1 (<1%) | 0 | 1 |
| *Prevotella multiformis* | - | Anaero | Oral flora, dental plaque | 1 (<1%) | 0 | 1 (1%) | 0 | 0 | 1 (<1%) | 0 | 1 |
| *Prevotella* sp. Oral clone BU035 | - | Anaero | Oral flora | 1 (<1%) | 0 | 0 | 0 | 0 | 0 | 1 (4%) | 0.11 |
| *Prevotella* sp. E7_34 E1 | - | Anaero | Oral flora, dental plaque | 1 (<1%) | 0 | 1 (1%) | 0 | 0 | 1 (<1%) | 0 | 1 |
| *Prevotella* sp. Oral clone F045 | - | Anaero | Dental plaque | 1 (<1%) | 0 | 1 (1%) | 0 | 0 | 1 (<1%) | 0 | 1 |
| *Prevotella veroralis* | - | Anaero | Oral flora, dental plaque | 1 (<1%) | 0 | 1 (1%) | 0 | 0 | 1 (<1%) | 0 | 1 |
| *Prevotellaceae bacterium* P4P_62 P1 | - | Anaero | Oral flora, dental plaque | 1 (<1%) | 0 | 1 (1%) | 0 | 0 | 1 (<1%) | 0 | 1 |
| *Propionibacterium acnes* | + | Anaero | Skin, oral flora, , gastrointestinal tract, urinary tract | 1 (<1%) | 1 (3%) | 0 | 0 | 0 | 1 (<1%) | 0 | 1 |
| *Pseudomonas fluorescens* | - | Aero | Soil, plants, water | 1 (<1%) | 0 | 1 (1%) | 0 | 0 | 1 (<1%) | 0 | 1 |
| *Pseudomonas mendocina* | - | Aero | Water , environment | 1 (<1%) | 0 | 1 (1%) | 0 | 0 | 1 (<1%) | 0 | 1 |
| *Ralstonia pickettii* | - | Aero | Environment | 1 (<1%) | 0 | 1 (1%) | 0 | 0 | 1 (<1%) | 0 | 1 |
| *Salmonella enterica subsp. Enterica* | - | Facultative | Water , red meat, reptiles | 1 (<1%) | 0 | 1 (1%) | 0 | 0 | 1 (<1%) | 0 | 1 |
| *Scardovia* genomosp. C1 | + | Anaero | Oral flora, dental plaque | 1 (<1%) | 1 (3%) | 0 | 0 | 0 | 1 (<1%) | 0 | 1 |
| *Selenomonas* sp. Oral clone GI064 | - | Anaero | Oral flora | 1 (<1%) | 0 | 1 (1%) | 0 | 0 | 1 (<1%) | 0 | 1 |
| *Selenomonas* sp. Oral clone P2PA_80 P4 | - | Anaero | Oral flora, dental plaque | 1 (<1%) | 1 (3%) | 0 | 0 | 0 | 1 (<1%) | 0 | 1 |
| *Selenomonas* sp. Oral clone FT050 | - | Anaero | Oral flora | 1 (<1%) | 0 | 0 | 0 | 1 (4%) | 1 (<1%) | 0 | 1 |
| *Serratia marcescens* | - | Facultative | Soil, water, plants | 1 (<1%) | 0 | 0 | 1 | 0 | 1 (<1%) | 0 | 1 |
| *Sphingobium yanoikuyae* | - | Aero | Soil, water, plants | 1 (<1%) | 0 | 0 | 0 | 0 | 0 | 1 (4%) | 0.11 |
| *Sphingomonas* sp. | - | Aero | Water, soil | 1 (<1%) | 0 | 1 (1%) | 0 | 0 | 1 (<1%) | 0 | 1 |
| *Staphylococcus cohnii* | + | Facultative | Skin | 1 (<1%) | 0 | 0 | 1 | 0 | 1 (<1%) | 0 | 1 |
| *Staphylococcus haemolyticus* | + | Facultative | Skin | 1 (<1%) | 0 | 0 | 0 | 1 (4%) | 1 (<1%) | 0 | 1 |
| *Staphylococcus hominis* | + | Facultative | Skin | 1 (<1%) | 0 | 0 | 0 | 1 (4%) | 1 (<1%) | 0 | 1 |
| *Lysinibacillus sphaericus* | + | Facultative | Soil, water | 1 (<1%) | 1 (3%) | 0 | 0 | 0 | 1 (<1%) | 0 | 1 |
| *Staphylococcus pasteuri* | + | Facultative | Water, food, environment | 1 (<1%) | 1 (3%) | 0 | 0 | 0 | 1 (<1%) | 0 | 1 |
| Uncultured *Abiotrophia* sp. Clone 401H03 | + | Facultative | Dental plaque | 1 (<1%) | 0 | 0 | 0 | 1 (4%) | 1 (<1%) | 0 | 1 |
| Uncultured *Actinomycetales bacterium* clone MFC-B162-F03 | ND | ND | Wastewater | 1 (<1%) | 0 | 0 | 0 | 1 (4%) | 1 (<1%) | 0 | 1 |
| Uncultured *Anaerococcus* sp. Clone ML2-55 | + | Anaero | Skin | 1 (<1%) | 0 | 0 | 0 | 0 | 0 | 1 (4%) | 0.11 |
| Uncultured *Arcobacter* sp. Clone DS126 | - | Facultative | Mangrove | 1 (<1%) | 0 | 0 | 0 | 1 (4%) | 1 (<1%) | 0 | 1 |
| Uncultured *Catonella* sp. Clone 402A04 | - | Anaero | Dental plaque | 1 (<1%) | 0 | 1 (1%) | 0 | 0 | 1 (<1%) | 0 | 1 |
| Uncultured *Eubacterium* sp. Clone 202E02 | + | Anaero | Dental plaque | 1 (<1%) | 0 | 0 | 0 | 1 (4%) | 1 (<1%) | 0 | 1 |
| Uncultured *Flavobacteriaceae bacterium* clone 4P2-62 | ND | ND | Skin | 1 (<1%) | 0 | 0 | 0 | 0 | 0 | 1 (4%) | 0.11 |
| Uncultured *Flavobacteriaceae bacterium* clone 4PN75 | ND | ND | Skin | 1(<1%) | 0 | 1 (1%) | 0 | 0 | 1 (1%) | 0 | 1 |
| Uncultured *Lautropia* sp. 202B04 | - | Facultative | Dental plaque | 1 (<1%) | 1 (3%) | 0 | 0 | 0 | 1 (<1%) | 0 | 1 |
| Uncultured *Neisseria* sp. Clone AV_4R-S-C15 | - | Aero | Environment | 1 (<1%) | 0 | 0 | 1 | 0 | 1 (<1%) | 0 | 1 |
| Uncultured *Neisseria* sp. Clone 401A08 | - | Aero | Dental plaque | 1 (<1%) | 0 | 1 (1%) | 0 | 0 | 1 (<1%) | 0 | 1 |
| Uncultured *Staphylococcus* sp. Clone Gcentralis14 | + | Facultative | Chewing lice of pocket gophers | 1 (<1%) | 0 | 1 (1%) | 0 | 0 | 1 (<1%) | 0 | 1 |
| Uncultured *Streptococcus* sp. Clone EHFS1 | + | Facultative | Environment | 1 (<1%) | 0 | 0 | 0 | 0 | 0 | 1 (4%) | 0.11 |
| Uncultured *Tannerella* sp. Clone 601H10 | - | Anaero | Dental plaque | 1 (<1%) | 0 | 1 (1%) | 0 | 0 | 1 (<1%) | 0 | 1 |

ND; not determined, *; no confirmed data.; CAP, community-associated pneumonia; VAP, ventilator-associated pneumonia; NV ICU-P, non-ventilator ICU pneumonia; AP, aspiration pneumonia; CS, control subjects.
