# Supplementary material for: Repertoire of Intensive Care Unit Pneumonia Microbiota
Source: PLoS One. 2012 Feb 28;7(2):e32486. doi: 10.1371/journal.pone.0032486 (PMC3289664; doi:10.1371/journal.pone.0032486)
Supplement: Table S5 — Molecular repertoire of fungi identified in the present study and their frequency in each cohort. (DOCX) [file pone.0032486.s013.docx]

Table S5: molecular repertoire of fungi identified in the present study and their frequency in each cohort

| Fungi | Origin | Frequency (n=210) | Frequency in pneumonia cohorts | | | | Frequency in pneumonia Vs CS | | |
| --- | --- | --- | --- | --- | --- | --- | --- | --- | --- |
|  |  |  | CAP (n=32) | VAP (n=106) | NV ICU-P (n=22) | AP (n=25) | Pneumonia patients (n=185) | CS (n=25) | P value |
| *Candida albicans* | Skin, oral cavity, gut flora, water, environment | 16 (8%) | 1 (3%) | 6 (6%) | 4 (20%) | 3 (12%) | 14 (8%) | 2 (8%) | 0.93 |
| *Candida parapsilosis* | Skin, oral cavity, water | 4 (2%) | 0 | 3 (3%) | 0 | 0 | 3 (2%) | 1 (4%) | 1 |
| *Candida glabrata* | Skin, oral cavity, environment | 3 (1%) | 0 | 1 (1%) | 1 (5%) | 1 (4%) | 3 (2%) | 0 | 0.41 |
| *Candida. Lusitaniae* | Skin, , urogenital tract, gastrointestinal tract | 3 (1%) | 0 | 2 (2%) | 0 | 0 | 2 (1%) | 1 (4%) | 0.24 |
| *Candida krusei* | Skin, gastrointestinal tract, water | 2 (1%) | 1 (3%) | 0 | 1 (5%) | 0 | 2 (1%) | 0 | 1 |
| *Candida dubliniensis* | Oral cavity , urogenital tract, gastrointestinal tract, feces | 2 (1%) | 0 | 2 (2%) | 0 | 0 | 2 (1%) | 0 | 1 |
| *Candida tropicalis* | Skin, oral cavity, water | 2 (1%) | 0 | 0 | 2 (9%) | 0 | 2 (1%) | 0 | 1 |
| *Candida utilis* | Wood surfaces | 2 (1%) | 0 | 0 | 0 | 0 | 0 | 2 (8%) | **0.01** |
| *Penicillium* sp. | Environment, soil | 2 (1%) | 1 (3%) | 0 | 0 | 1 (4%) | 2 (1%) | 0 | 1 |
| *Saccharomyces cerevisiae* | Skins of grapes | 2 (1%) | 1 (3%) | 1 (1%) | 0 | 0 | 2 (1%) | 0 | 1 |
| *Aspergillus fumigatus* | Soil, environment, | 1 (<1%) | 0 | 1 (1%) | 0 | 0 | 1 (<1%) | 0 | 1 |
| *Aspergillus* sp. | Soil, plant debris, wood, environment | 1 (<1%) | 1 (3%) | 0 | 0 | 0 | 1 (<1%) | 0 | 1 |
| *Candida atlantica* | Water | 1 (<1%) | 0 | 1 (1%) | 0 | 0 | 1 (<1%) | 0 | 1 |
| *Candida kefyr* | Milk products | 1 (<1%) | 0 | 0 | 1 (5%) | 0 | 1 (<1%) | 0 | 1 |
| *Cladosporium sphaerospermum* | Soil, environment foodstuffs, hypersaline water | 1 (<1%) | 0 | 1 (1%) | 0 | 0 | 1 (<1%) | 0 | 1 |
| *Cladosporium* sp. | Soil, wood, environment foodstuffs, | 1 (<1%) | 1 (3%) | 0 | 0 | 0 | 1 (<1%) | 0 | 1 |
| *Cladophialophora boppii* | Soil | 1 (<1%) | 1 (3%) | 0 | 0 | 0 | 1 (<1%) | 0 | 1 |
| *Cryptococcus victoriae* | Environment, soil | 1 (<1%) | 0 | 0 | 1 (5%) | 0 | 1 (<1%) | 0 | 1 |
| *Davidiella tassiana* | Soil, environment | 1 (<1%) | 0 | 0 | 1 (5%) | 0 | 1 (<1%) | 0 | 1 |
| *Hyphoderma praetermissum* | Wood | 1 (<1%) | 0 | 1 (1%) | 0 | 0 | 1 (<1%) | 0 | 1 |
| *Melanized limestone ascomycete* CR-2004 | Limestone, building surface | 1 (<1%) | 0 | 1 (1%) | 0 | 0 | 1 (<1%) | 0 | 1 |
| *Mycosphaerella* sp. | Plants, salt marsh vegetation, environment | 1 (<1%) | 1 (3%) | 0 | 0 | 0 | 1 (<1%) | 0 | 1 |
| *Periconia macrospinosa* | Soil, environment | 1 (<1%) | 0 | 0 | 0 | 0 | 0 | 1 (4%) | 0.11 |
| *Sporidiobolales* sp. LM538 | Sea Water | 1 (<1%) | 1 (3%) | 0 | 0 | 0 | 1 (<1%) | 0 | 1 |

CAP, community-associated pneumonia; VAP, ventilator-associated pneumonia; NV ICU-P, non-ventilator ICU pneumonia; AP, aspiration pneumonia; CS, control subjects.
