# Supplementary material for: Repertoire of Intensive Care Unit Pneumonia Microbiota
Source: PLoS One. 2012 Feb 28;7(2):e32486. doi: 10.1371/journal.pone.0032486 (PMC3289664; doi:10.1371/journal.pone.0032486)
Supplement: Table S6 — Viruses identified by qPCR and their frequency in each cohort. (DOCX) [file pone.0032486.s014.docx]

**Table S6: viruses identified by qPCR and their frequency in each cohort**

| Viruses | Family | Frequency (n=210) | Frequency in pneumonia cohorts | | | | Frequency in pneumonia Vs CS | | |
| --- | --- | --- | --- | --- | --- | --- | --- | --- | --- |
|  |  |  | CAP (n=32) | VAP (n=106) | NV ICU-P (n=22) | AP (n=25) | Pneumonia patients (n=185) | CS (n=25) | P value |
| HSV | Herpesviridae | 58 (28%) | 7 (22%) | 32 (30%) | 8 (36%) | 4 (16%) | 51 (28%) | 7 (28%) | 0.96 |
| CMV | Herpesviridae | 34 (16%) | 5 (16%) | 21 (20%) | 5 (23%) | 0 | 31 (17%) | 3 (12%) | 0.54 |
| PIV-1 | Paramyxoviridae | 4 (2%) | 0 | 3 (3%) | 0 | 0 | 3 (2%) | 1 (4%) | 0.41 |
| VZV | Herpesviridae | 3 (1%) | 1 (3%) | 2 (2%) | 0 | 0 | 3 (2%) | 0 | 1 |
| Coronavirus OC43 | Coronaviridae | 2 (1%) | 0 | 1 (1%) | 0 | 1 (4%) | 2 (1%) | 0 | 1 |
| Rhinovirus | Picornaviridae | 1 (<1%) | 0 | 0 | 0 | 0 | 0 | 1 (4%) | 0.09 |
| RSV-A | Paramyxoviridae | 1 (<1%) | 0 | 0 | 1 (5%) | 0 | 1 (<1%) | 0 | 1 |

CAP, community-associated pneumonia; VAP, ventilator-associated pneumonia; NV ICU-P, non-ventilator ICU pneumonia; AP, aspiration pneumonia; CS, control subjects.
