# Supplementary material for: Repertoire of Intensive Care Unit Pneumonia Microbiota
Source: PLoS One. 2012 Feb 28;7(2):e32486. doi: 10.1371/journal.pone.0032486 (PMC3289664; doi:10.1371/journal.pone.0032486)
Supplement: Table S7 — Clinical data in monobacterial, polybacterial, fungal and/or viral and sterile episodes. (DOCX) [file pone.0032486.s015.docx]

**Table S7: clinical data in monobacterial, polybacterial, fungal and/or viral and sterile episodes**

|  | *Monobacterial BAL* | *Polybacterial BAL with dominant bacterium* | *Polybacterial BAL without dominant bacterium* | *BAL with viruses or/and fungi only* | *Sterile BAL* |
| --- | --- | --- | --- | --- | --- |
| Pneumonia patients (n=135/185) |  |  |  |  |  |
| Case number | 39 | 27 | 29 | 19 | 21 |
| Age, yr (SD) | 62.5 (14.3) | 60.3 (15.8) | 60.8 (15.4) | 63.9 (12.8) | 56.1 (14.4) |
| Male gender | 22 | 10 | 20 | 10 | 11 |
| Female gender | 17 | 17 | 9 | 9 | 10 |
| Immunocompromized | 11 | 10 | 5 | 9 | 7 |
| ARDS | 12 | 4 | 6 | 12 | 11 |
| CPIS (SD) | 3.7 (1.9) | 3.7 (1.5) | 3.3 (1.6) | 3.7 (1.9) | 4.1 (1.8) |
| SOFA score (SD) | 6.6 (3.2) | 7.1 (3.8) | 5.8 (3.1) | 8.2 (3.6) | 7.7 (3.3) |
| Radiologic score (SD) | 4.6 (2.0) | 4.4 (2.9) | 5.1 (3.3) | 6.6 (3.1) | 5.5 (3.6) |
| Temperature, °C (SD) | 37.8 (1.1) | 37.5 (2.8) | 38.0 (0.8) | 37.3 (0.9) | 37.6 (1.4) |
| PaO2/FIO2 (SD) | 206 (89) | 200 (75) | 199 (79) | 153 (86) | 157 (61) |
| Initial antibiotic therapy | 18 | 12 | 11 | 10 | 12 |
| Less than 2 days prior to pneumonia | 9 | 9 | 4 | 1 | 7 |
| 3 days or more prior to pneumonia | 9 | 3 | 7 | 9 | 5 |
| Length of ICU stay prior to pneumonia, d (SD) | 9.0 (11.6) | 3.6 (4.7) | 8.0 (14.2) | 5.6 (5.3) | 4.4 (4.3) |
| Length of hospital stay, d (SD) | 28.3 (33.0) | 19.1 (17.3) | 30.0 (26.5) | 23.6 (14.5) | 24.6 (18.5) |
| Sepsis | 9 | 3 | 8 | 6 | 5 |
| Septic shock | 20 | 9 | 14 | 10 | 7 |
| ICU mortality (%) | 13 (33) | 4 (14) | 11 (37) | 11 (57) | 4 (19) |
| Control subjects (n=22/25) |  |  |  |  |  |
| Case number | 1 | 4 | 7 | 6 | 4 |
| Age, yr | 85 | 60.7 (17.2) | 47.2 (14) | 62.8 (18.7) | 49.2 (26.9) |
| Male gender | 1 | 3 | 2 | 4 | 3 |
| Female gender | 0 | 1 | 5 | 2 | 1 |
| Immunocompromized | 0 | 1 | 3 | 1 | 1 |
| ARDS | 0 | 1 | 2 | 1 | 0 |
| CPIS (SD) | 4 | 5 (1.4) | 3.8 (1.2) | 4.5 (1.2) | 2.2 (1.7) |
| SOFA score (SD) | 8 | 4 (2.1) | 6.1 (3.0) | 5.6 (2.9) | 6.5 (1.2) |
| Temperature, °C (SD) | 38.5 | 37.1 (1.9) | 37.8 (1.2) | 37.4 (1.3) | 37.3 (0.5) |
| Ventilated patients | 1 | 3 | 4 | 4 | 4 |
| Length of MV prior to sampling, d (SD) | 1 | 10 (9.8) | 5.7 (6.2) | 5.5 (8.3) | 1 (0.8) |
| Initial antibiotic therapy | 1 | 4 | 4 | 1 | 4 |
| Less than 2 days prior to sampling | 0 | 1 | 2 | 0 | 2 |
| 3 days or more prior to sampling | 1 | 3 | 2 | 1 | 2 |
| Length of ICU stay prior to sampling, d (SD) | 1 | 5.7 (7.5) | 14.8 (18.2) | 12.6 (16.9) | 1.5 (0.5) |
| Total length of hospital stay, d (SD) | 3 | 43 (42.5) | 22.3 (24.2) | 36.2 (44) | 9.25 (3.8) |
| Developed pneumonia after control sampling | 0 | 2 | 0 | 2 | 0 |
| Sepsis | 0 | 2 | 0 | 1 | 0 |
| Septic shock | 0 | 2 | 2 | 4 | 2 |
| ICU mortality (%) | 0 | 1 (25) | 1 (14) | 1 (16) | 2 (50) |

(SD), Standard Deviation
