# Supplementary material for: Repertoire of Intensive Care Unit Pneumonia Microbiota
Source: PLoS One. 2012 Feb 28;7(2):e32486. doi: 10.1371/journal.pone.0032486 (PMC3289664; doi:10.1371/journal.pone.0032486)
Supplement: Table S8 — Comparison between culture and molecular assays performed on BAL specimens. (DOCX) [file pone.0032486.s016.docx]

Table S8: comparison between culture and molecular assays performed on BAL specimens

| *Molecular results* | *Culture results* | |
| --- | --- | --- |
|  | Positive | Negative |
| CAP (n=32) |  |  |
| Positive | 14 | 11 |
| Negative | 3 | 4 |
| VAP (n=106) |  |  |
| Positive | 79 | 14 |
| Negative | 7 | 6 |
| NV-ICU-P (n=22) |  |  |
| Positive | 17 | 3 |
| Negative | 1 | 2 |
| AP (n=25) |  |  |
| Positive | 15 | 3 |
| Negative | 2 | 5 |
| CS (n=25) |  |  |
| Positive | 14 | 6 |
| Negative | 3 | 2 |

CAP, community-associated pneumonia; VAP, ventilator-associated pneumonia; NV ICU-P, non-ventilator ICU pneumonia; AP, aspiration pneumonia; CS, control subjects.
