# Supplementary material for: Repertoire of Intensive Care Unit Pneumonia Microbiota
Source: PLoS One. 2012 Feb 28;7(2):e32486. doi: 10.1371/journal.pone.0032486 (PMC3289664; doi:10.1371/journal.pone.0032486)
Supplement: Table S9 — Clinical and sociodemographic data of patients and controls. (DOCX) [file pone.0032486.s017.docx]

Table S9: clinical and sociodemographic data of patients and controls

|  | *CAP (n=32)* | *VAP (n=106)* | *NV ICU-P (n=22)* | *AP (n=25)* | *Pneumonia patients (n=185)* | *CS (n=25)* |
| --- | --- | --- | --- | --- | --- | --- |
| Sex |  |  |  |  |  |  |
| Male (%) | 20 (62%) | 60 (57%) | 16 (73%) | 10 (40%) | 106 (57%) | 15 (60%) |
| Female (%) | 12 (38%) | 46 (43%) | 6 (27%) | 15 (60%) | 79 (43%) | 10 (40%) |
| Age |  |  |  |  |  |  |
| Age range (years) | 23 to 94 | 18 to 85 | 32 to 84 | 22 to 85 | 18 to 94 | 18 to 85 |
| Age (mean ± SD years) | 60.62 ± 16.27 | 57.81 ± 15.26 | 61.54 ± 13.32 | 58.16 ± 17.17 | 58.78 ± 15.43 | 56.72 ± 19.15 |
| Immunosupression (%) | 12 (38%) | 42 (40%) | 11 (50%) | 9 (36%) | 74 (40%) | 4 (16%) |
| ARDS (%) | 12 (38%) | 35 (33%) | 9 (41%) | 3 (12%) | 59 (31%) | 7 (28%) |

ARDS, Acute respiratory distress syndrome; CAP, community-associated pneumonia; VAP, ventilator-associated pneumonia; NV ICU-P, non-ventilator ICU pneumonia; AP, aspiration pneumonia; CS, control subjects.
