# Supplementary material for: Repertoire of Intensive Care Unit Pneumonia Microbiota
Source: PLoS One. 2012 Feb 28;7(2):e32486. doi: 10.1371/journal.pone.0032486 (PMC3289664; doi:10.1371/journal.pone.0032486)
Supplement: Table S10 — Repertoire of bacteria identified by culture and their frequency in each cohort. (DOCX) [file pone.0032486.s018.docx]

**Table S10: repertoire of bacteria identified by culture and their frequency in each cohort**

| *Bacteria* | *Gram* | *Aero/Anaero* | *Origin* | *Frequency* | | | | | *Previously reported in pneumonia* |
| --- | --- | --- | --- | --- | --- | --- | --- | --- | --- |
|  |  |  |  | CAP (n=32) | VAP (n=106) | NV ICU-P (n=22) | AP (n=25) | CS (n=25) |  |
| *Staphylococcus aureus* | + | Facultative | Skin, nasopharyngeal mucosa | 2 | 11 | 3 | 8 | 2 | Yes (frequently) |
| *Pseudomonas aeruginosa* | - | Aero | Water, gastrointestinal tract, environment, oral flora | 3 | 16 | 4 | 1 | 1 | Yes (frequently) |
| *Klebsiella pneumoniae* | - | Facultative | Water, soil, environment | 0 | 6 | 2 | 0 | 0 | Yes (rarely) |
| *Escherichia coli* | - | Aero | Water , feces, gastrointestinal tract | 1 | 3 | 0 | 3 | 0 | Yes (frequently) |
| *Haemophilus influenzae* | - | Facultative | Oral flora, dental plaque | 3 | 3 | 1 | 0 | 0 | Yes (frequently) |
| *Streptococcus pneumoniae* | + | Facultative | Nasopharyngeal Mucosa | 3 | 1 | 1 | 0 | 0 | Yes (frequently) |
| *Stenotrophomonas maltophilia* | - | Aero | Water, Soil | 0 | 3 | 2 | 0 | 0 | Yes |
| *Staphylococcus epidermidis* | + | Facultative | Skin, Nasopharyngeal Mucosa | 0 | 2 | 1 | 0 | 0 | Yes |
| *Achromobacter xilosoxidans* | - | Aero | Water, environment | 0 | 1 | 0 | 0 | 1 | Yes (rarely) |
| *Proteus mirabilis* | - | Facultative | Soil, water, gastrointestinal tract | 0 | 2 | 0 | 0 | 0 | Yes |
| *Serratia marcescens* | - | Facultative | Soil, water, plants | 0 | 1 | 1 | 0 | 0 | Yes |
| *Klebsiella oxytoca* | - | Facultative | Water, soil, environment | 0 | 1 | 0 | 0 | 1 | Yes (rarely) |
| *Providencia stuartii* | - |  | Soil, water, sewage, gastrointestinal tract | 0 | 2 | 0 | 0 | 0 | Yes (rarely) |
| *Citrobacter koseri* | - | Facultative | Gastrointestinal tract, feces, Water, soil | 0 | 2 | 0 | 0 | 0 | Yes (rarely) |
| *Acinetobacter baumannii* | - | Aero | Water, soil | 0 | 2 | 0 | 0 | 0 | Yes |
| *Enterobacter aerogenes* | - | Facultative | Water, soil, gastrointestinal tract | 0 | 1 | 1 | 0 | 0 | Yes (frequently) |
| *Branhamella catarrhalis* | - | Aero | Oral flora, Nasopharyngeal Mucosa | 1 | 0 | 0 | 0 | 0 | Yes (frequently) |
| *Enterococcus faecalis* | + | Facultative | Soil,, water, intestinal flora | 0 | 1 | 0 | 0 | 0 | Yes (rarely) |
| *Raoultella ornithinolytica* | - | Facultative | Water, soil, plants | 0 | 1 | 0 | 0 | 0 | Yes (rarely) |
| *Corynebacterium striatum* | + | Facultative | Skin flora | 0 | 1 | 0 | 0 | 0 | Yes (rarely) |
| *Enterococcus faecium* | + | Facultative | Gastrointestinal and vaginal tract, oral flora, Water, soil | 0 | 0 | 1 | 0 | 0 | Yes (rarely) |
| *Morganella morganii* | - | Facultative | Gastrointestinal tract | 0 | 1 | 0 | 0 | 0 | Yes (rarely) |
| *Enterobacter cloacae* | - | Facultative | Skin, plant, water, soil, gastrointestinal tract | 0 | 1 | 0 | 0 | 0 | Yes |

**CAP, community-associated pneumonia; VAP, ventilator-associated pneumonia; NV ICU-P, non-ventilator ICU pneumonia; AP, aspiration pneumonia; CS, control subje**
