# Supplementary material for: Repertoire of Intensive Care Unit Pneumonia Microbiota
Source: PLoS One. 2012 Feb 28;7(2):e32486. doi: 10.1371/journal.pone.0032486 (PMC3289664; doi:10.1371/journal.pone.0032486)
Supplement: Table S11 — Repertoire of fungi identified by culture and their frequency in each cohort. (DOCX) [file pone.0032486.s019.docx]

Table S11: repertoire of fungi identified by culture and their frequency in each cohort

| *Fungi* | *Origin* | *Frequency* | | | | | *Previously reported in pneumonia* |
| --- | --- | --- | --- | --- | --- | --- | --- |
|  |  | CAP (n=32) | VAP (n=106) | NV ICU-P (n=22) | AP (n=25) | CS (n=25) |  |
| *Candida albicans* | Skin, oral cavity, gut flora, water, environment | 6 | 32 | 6 | 8 | 4 | Yes (frequently) |
| *Candida glabrata* | Skin, oral cavity, environment | 2 | 5 | 2 | 2 | 1 | Yes |
| *Aspergillus fumigatus* | Soil, environment, | 2 | 5 | 1 | 1 | 0 | Yes |
| *Candida tropicalis* | Skin, oral cavity, water | 1 | 4 | 0 | 1 | 1 | Yes |
| *Candida krusei* | Skin, gastrointestinal tract, water | 1 | 5 | 1 | 1 | 0 | Yes |
| *Candida parapsilosis* | Skin, oral cavity, water | 0 | 5 | 0 | 0 | 1 | Yes |
| *Candida lusitaniae* | Skin, , urogenital tract, gastrointestinal tract | 2 | 3 | 0 | 0 | 0 | Yes (rarely) |
| *Geotrichum* sp | Environment, water, soil, milk products, plants, gastrointestinal tract | 0 | 1 | 0 | 0 | 1 | Yes (rarely) |
| *Aspergillus niger* | Environment, soil, plants, milk products | 0 | 2 | 1 | 0 | 0 | Yes (rarely) |
| *Candida. zemplinina* | wine grapes | 2 | 0 | 0 | 0 | 0 | No |
| *Pichia kluyveri* | Grape must, plants | 2 | 0 | 0 | 0 | 0 | No |
| *Candida. inconspicua* | Skin, oral cavity, milk products | 2 | 0 | 0 | 0 | 0 | No |
| *Candida kefyr* | Milk products | 0 | 1 | 0 | 0 | 0 | Yes (rarely) |
| *Candida utilis* | Wood surfaces | 0 | 0 | 0 | 0 | 1 | No |
| *Candida dubliniensis* | Oral cavity , urogenital tract, gastrointestinal tract, feces | 0 | 1 | 0 | 0 | 0 | Yes (rarely) |
| *Candida rugosa* |  | 0 | 1 | 0 | 0 | 0 |  |
| *Saccharomyces cerevisiae* | Skins of grapes | 0 | 1 | 0 | 0 | 0 | Yes |
| *Penicillium* sp | Environment, soil | 0 | 0 | 0 | 0 | 1 | Yes |
| *Cladosporium* sp | Soil, wood, environment foodstuffs, | 0 | 0 | 0 | 0 | 1 | Yes |

**CAP, community-associated pneumonia; VAP, ventilator-associated pneumonia; NV ICU-P, non-ventilator ICU pneumonia; AP, aspiration pneumonia; CS, control subje**
