# Supplementary material for: Repertoire of Intensive Care Unit Pneumonia Microbiota
Source: PLoS One. 2012 Feb 28;7(2):e32486. doi: 10.1371/journal.pone.0032486 (PMC3289664; doi:10.1371/journal.pone.0032486)
Supplement: Table S12 — Repertoire of bacteria identified by blood culture and their frequency in each cohort. (DOCX) [file pone.0032486.s020.docx]

Table S12: repertoire of bacteria identified by blood culture and their frequency in each cohort

| *Bacteria* | *Gram* | *Aero/Anaero* | *Origin* | *Frequency* | | | | | *Previously reported in pneumonia* |
| --- | --- | --- | --- | --- | --- | --- | --- | --- | --- |
|  |  |  |  | CAP (n=32) | VAP (n=106) | NV ICU-P (n=22) | AP (n=25) | CS (n=25) |  |
| *Staphylococcus aureus* | + | Facultative | Skin, nasopharyngeal mucosa | 1 | 3 | 1 | 3 | 0 | Yes (frequently) |
| *Staphylococcus epidermidis* | + | Facultative | Skin, nasopharyngeal mucosa | 0 | 3 | 2 | 0 | 0 | Yes |
| *Pseudomonas aeruginosa* | - | Aero | Water, gastrointestinal tract, environment, oral flora | 1 | 3 | 0 | 0 | 0 | Yes (frequently) |
| *Escherichia coli* | - | Aero | Water , feces, gastrointestinal tract | 0 | 2 | 0 | 0 | 0 | Yes (frequently) |
| *Staphylococcus hominis* | + | Facultative | Skin | 0 | 1 | 0 | 0 | 1 | Yes (rarely) |
| *Sphingomonas paucimobilis* | - | Aero | Water, soil, environment | 0 | 1 | 0 | 0 | 0 | Yes (rarely) |
| *Staphylococcus warneri* | + | Facultative | Skin flora | 0 | 0 | 0 | 1 | 0 | Yes (rarely) |
| *Stenotrophomonas maltophilia* | - | Aero | Water, Soil | 0 | 1 | 0 | 0 | 0 | Yes |
| *Klebsiella pneumoniae* | - | Facultative | Water, soil, environment | 0 | 0 | 1 | 0 | 0 | Yes (rarely) |
| *Enterococcus faecalis* | + | Facultative | Soil,, water, intestinal flora | 0 | 1 | 0 | 0 | 0 | Yes (rarely) |
| *Serratia marcescens* | - | Facultative | Soil, water, plants | 0 | 0 | 1 | 0 | 0 | Yes |
| *Streptococcus mitis* | + | Facultative | Oral flora, dental plaque | 0 | 0 | 1 | 0 | 0 | Yes (rarely) |
| *Enterobacter aerogenes* | - | Facultative | Water, soil, gastrointestinal tract | 0 | 1 | 0 | 0 | 0 | Yes (frequently) |
| *Proteus mirabilis* | - | Facultative | Soil, water, gastrointestinal tract | 0 | 0 | 0 | 0 | 1 | Yes |

**CAP, community-associated pneumonia; VAP, ventilator-associated pneumonia; NV ICU-P, non-ventilator ICU pneumonia; AP, aspiration pneumonia; CS, control subje**
