# Supplementary material for: Repertoire of Intensive Care Unit Pneumonia Microbiota
Source: PLoS One. 2012 Feb 28;7(2):e32486. doi: 10.1371/journal.pone.0032486 (PMC3289664; doi:10.1371/journal.pone.0032486)
Supplement: Table S13 — Microorganisms identified by serologic testing and their frequency in each cohort. (DOCX) [file pone.0032486.s021.docx]

Table S13: microorganisms identified by serologic testing and their frequency in each cohort

| *Serology* | *Frequency* | | | | | | | | | |
| --- | --- | --- | --- | --- | --- | --- | --- | --- | --- | --- |
|  | CAP (n=32) | | VAP (n=106) | | NV ICU-P (n=22) | | AP (n=25) | | CS (n=25) | |
|  | Definite | Lowly positive | Definite | Lowly positive | Definite | Lowly positive | Definite | Lowly positive | Definite | Lowly positive |
| Bacteria |  |  |  |  |  |  |  |  |  |  |
| *Coxiella burnetii* | 0 | 0 | 0 | 1 | 1 | 0 | 1 | 0 | 0 | 0 |
| Viruses |  |  |  |  |  |  |  |  |  |  |
| HSV (IgM) | 2 | 4 | 3 | 10 | 0 | 0 | 0 | 2 | 1 | 3 |
| CMV (IgM) | 1 | 1 | 9 | 4 | 1 | 0 | 0 | 0 | 1 | 0 |
| CMV (Antigenemia) | 1 | 3 | 4 | 3 | 0 | 2 | 0 | 0 | 0 | 1 |
| VZV (IgM) | 1 | 3 | 3 | 6 | 0 | 1 | 1 | 0 | 2 | 1 |
| Influenza virus A | 1 | 0 | 4 | 0 | 0 | 0 | 0 | 0 | 0 | 0 |
| Fungi |  |  |  |  |  |  |  |  |  |  |
| *Aspergillus* (ELISA) | 3 | 1 | 11 | 12 | 4 | 2 | 1 | 1 | 3 | 4 |

CAP, community-associated pneumonia; VAP, ventilator-associated pneumonia; NV ICU-P, non-ventilator ICU pneumonia; AP, aspiration pneumonia; CS, control subject
